# New 1,2,3-Triazole-Containing Hybrids as Antitumor Candidates: Design, Click Reaction Synthesis, DFT Calculations, and Molecular Docking Study

**DOI:** 10.3390/molecules26030708

**Published:** 2021-01-29

**Authors:** Islam H. El Azab, Hamdy S. El-Sheshtawy, Rania B. Bakr, Nadia A. A. Elkanzi

**Affiliations:** 1 Chemistry Department, College of Science, Taif University, P.O. Box 11099, Taif 21944, Saudi Arabia; 2 Chemistry Department, Faculty of Science, Aswan University, P.O. Box 81528 Aswan, Egypt; nahasan@ju.edu.sa; 3 Chemistry Department, Faculty of Science, Kafrelsheikh University, Kafr ElSheikh 33516, Egypt; h.elsheshtawy@sci.kfs.edu.eg; 4 Department of Pharmaceutical Chemistry, College of Pharmacy, Jouf University, P.O. Box 2014 Sakaka, Saudi Arabia; rbbakr@ju.edu.sa; 5 Department of Pharmaceutical Organic Chemistry, Faculty of Pharmacy, Beni-Suef University, Beni-Suef 62514, Egypt; 6 Chemistry Department, College of Science, Jouf University, P.O. Box 2014 Sakaka, Saudi Arabia

**Keywords:** click chemistry, cycloaddition reaction, pyrazole, 1,2,3-triazole, molecular docking, antitumor activity, DFT calculation

## Abstract

In an effort to improve and achieve biologically active anticancer agents, a novel series of 1,2,3-triazole-containing hybrids were designed and efficiently synthesized *via* the Cu-catalyzed azide-alkyne cycloaddition (CuAAC) reaction of substituted-arylazides with alkyne-functionalized pyrazole-[1,2,4]-triazole hybrids. The structure geometry of these new clicked 1,2,3-triazoles was explored by density functional theory (DFT) using the B3LYP/6-311++G(d,p) level; also, the potential activity of the compounds for light absorption was simulated by time-dependent DFT calculations (TD-DFT). The antitumor impacts of the newly synthesized compounds were *in vitro* estimated to be towards the human liver cancer cell line (HepG-2), the human colon cancer cell line (HCT-116), and human breast adenocarcinoma (MCF-7). Among the tested compounds, conjugate **7** was the most potent cytotoxic candidate towards HepG-2, HCT-116, and MCF-7, with IC_50_ = 12.22, 14.16, and 14.64 µM, respectively, in comparison to that exhibited by the standard drug doxorubicin (IC_50_ = 11.21, 12.46, and 13.45 µM). Finally, a molecular docking study was conducted within the epidermal growth factor receptor (EGFR) active site to suggest possible binding modes. Hence, it could conceivably be hypothesized that analogies **7**, **6,** and **5** could be considered as decent lead candidate compounds for anticancer agents.

## 1. Introduction

**Figure 1:**
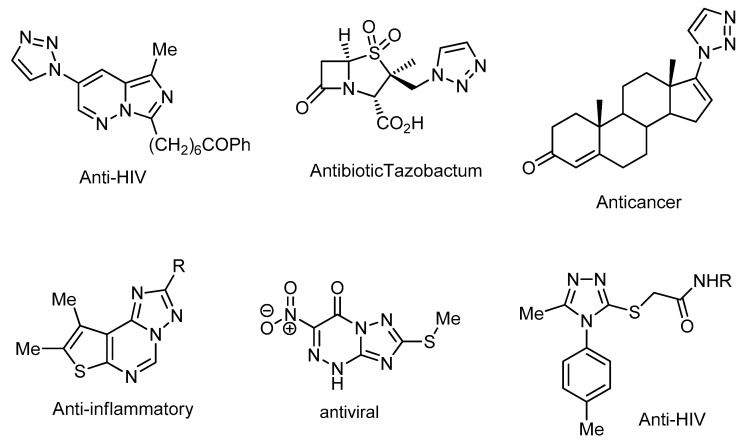
Representative samples of bioactive triazoles.

According to the most recent report from the universal health organization, cancer is the second most common cause of death across the world, with more than 9.6 million deaths a year. In the last few years, numerous anticancer drugs have been effectively improved. Nevertheless, most of the developed anti-cancer drugs are not very effective, and side effects might concurrently occur, such as drug-induced impedance. Therefore, it is crucial to discover and improve novel alternative safe and effective drugs with fewer side effects for the long-term treatment of cancer [1,2]. The basic unit existing in a numerous medicinal drug is mainly 1,2,3-triazole and/or 1,2,4-triazole moieties (Figure 1), and their analogies have attracted endless interest in the field of medicinal chemistry. Triazole-containing heterocycles are architectures used for the construction of lead molecules, attracting the interest of researchers owing to their wide range of biological impacts, especially as anticancer [3], antimicrobial [4], anti-tubercular [5], anticonvulsant [6], antibacterial [7], anti-inflammatory [8], analgesic [9], antiviral [10], and anti-HIV agents [11]. 

1,2,3-triazoles, as significant heterocyclic platforms, can be easily produced in an excellent yield by the click reaction of alkynes and azido derivatives; this reaction has value due to the ease with which alkynes and azides can be merged into a single architecture. The vast scope of the copper(I)-catalyzed azide alkyne cycloaddition (CuAAC), is demonstrated *via* its usage in various fields of life and material sciences, such as in drug discovery [12], oligonucleotide synthesis, [13] and DNA labeling [14]. 

The progress in click chemistry can clearly be observed recently, as numerous synthetic approaches for formulating 1,2,3-triazole scaffolds have been reported. Several reviews have reported the click reaction as essential in numerous fields—for instance, in polymer grafting [15], in the synthesis of 1,2,3-triazole scaffolds [16], and in chemical ligation [17]. Recently, reported works have declared applications in the achievement of peptidomimetics [18], in surface chemistry [19], and in bio-conjugations [20].

Based on these applications of triazole motifs and as extension of our efforts in the synthesis and modulation of azoles as a new anticancer agent [21,22,23,24,25,26,27], we report herein the annulation of novel pharmacophore architectures *via* the integration of small chemical moieties, pyrazole and 1,2,3-triazole, of significant pharmacophoric impact, with aim of developing their therapeutic impacts.

## 2. Results and Discussion

### 2.1. Chemistry

**Scheme 1:**
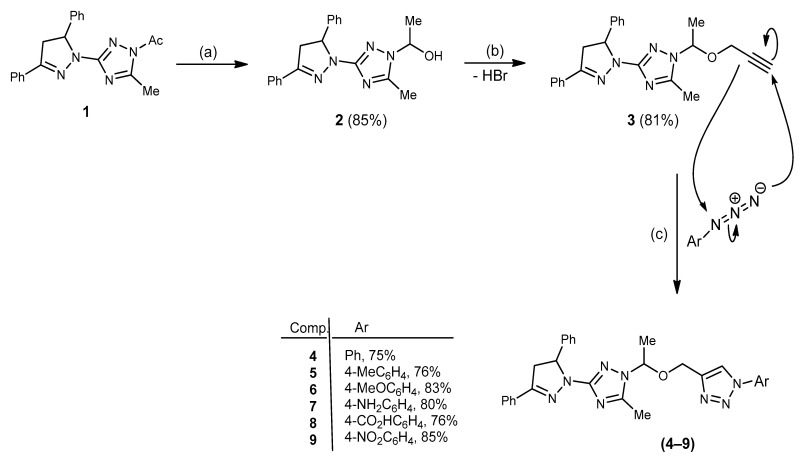
*Reagents and conditions*: (**a**) NaBH_4_, dry MeOH, stirring, rt, 1 h; (**b**) propargyl bromide, DMF, K_2_CO_3_, reflux/ stirring, 6 h; (**c**) Na-ascorbate, CuSO_4_·5H_2_O, DMF: H_2_O (6:1), stirring, 60–70 °C, 5–7 h.

The synthetic routes for the annulation of the precursors and title compounds are outlined in Scheme 1. Herein, a series of *N*-(4-substituted phenyl)-4-((1-(3-(3,5-diphenyl-4,5-dihydro-1*H*-pyrazol-1-yl)-5-methyl-1*H*-1,2,4-triazol-1-yl)ethoxy)methyl)-1*H*-1,2,3-triazoles (**4**–**9**) were annulated with the aim of exploring the effect of the substitution in the phenyl moiety on their antitumor impacts. The required starting compound, *N*-acetyl 1,2,4-triazole analogue **1**, was prepared as per our reported work [26]. This compound **1** was transformed into the corresponding secondary alcohol **2** *via* reduction with NaBH_4_ in dry MeOH. The *O*-alkylation of compound **2** with propargyl bromide in DMF containing K_2_CO_3_ yielded the propargyl analogue **3** in an 81% yield, as can be seen in Scheme 1. Finally, the propargyl substrate **3** was clicked with various aromatic azides in DMF containing catalytic Na-ascorbate to furnish 1-(4-substituted phenyl)-1*H*-1,2,3-triazole conjugates (**4**–**9**) in 75–85% yields. The structure of 1,2,3-triazole conjugates **4**–**9** was simply elucidated using spectral and elemental tools. Their IR spectra disclosed the lack of acetylenic bond stretching, which was originally observed in compound **3** (IR) at 2120 cm^−1^; meanwhile, the C−O−C linkage stretching band at 1067 cm^−1^ was still observed. Moreover, the ^1^H-NMR spectra disclosed the lack of acetylenic proton resonance initially detected in compound **3** (^1^H-NMR) at 3.20 ppm; in addition, new singlet resonances at ≈ 8.11 ppm were attributed to the 1,2,3-triazole-H5 proton, whereas the (^13^C-NMR) spectra of these conjugates revealed six singlet resonances in the aliphatic region at ≈ 11.0, 20.0 (2Me); 40.1, 55.4 (2CH_2_); and 59.7, 83.7 (2CH) ppm. For each compound, its mass spectrum revealed a molecular ion peak that corresponded to its molecular formula. 

### 2.2. DFT Calculations

**Figure 2:**
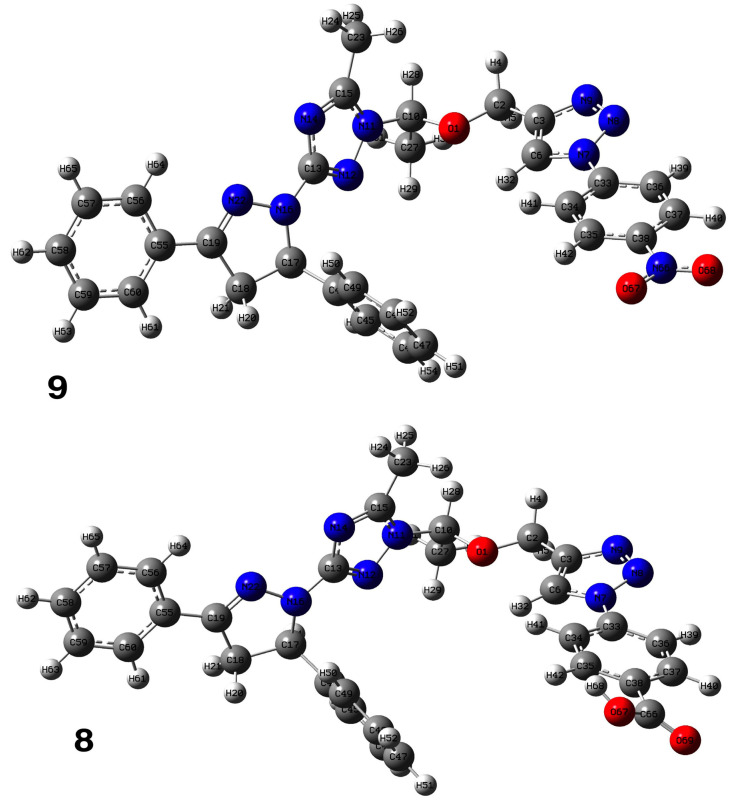
Optimized structure of **8** and **9** derivatives at the B3LYP/6-311++G(d,p) level of theory in the gas phase.

The structure and geometry of the triazole conjugates (**4**–**9**) were investigated by the DFT (B3LYP/6-311++G(d,p)) method. The substituent on the phenyl group modulated the geometry of the (**4**–**9**) triazole derivatives. A triazole ring bearing the N7-N8-N9 moiety played a significant role in the binding with epidermal growth factor receptor (EGFR) (see molecular docking analysis). The presence of intramolecular hydrogen bonding at C38H39…N8 with the bond length of 2.57, 2.58, 2.64, 2.62, 2.52, and 2.48 Å for **4**, **5**, **6**, **7**, **8**, and **9**, respectively, twisted the terminal group and deviated from linearity. According to the HB bond distance, the strength of the intramolecular H-bonding was in the order **9** > **8** > **4** > **5** > **7** > **6**. Hence, the short hydrogen bonding (C38H39…N8) distance deteriorates the binding of triazole derivatives with EGFR (see docking results). The dihedral angle between substituted phenyl groups was 150° for unsubstituted phenyl (**4**) and the *p*-tolyl (**5**) derivatives (Figure S1). The dihedral angle decreased to 144° and 146° for the electron-donating substituents derivatives Me-O (**6**) and NH_2_ (**7**), respectively (Figure S2). On the other hand, the dihedral angle increased to 156° and 163° in the electron-pulling groups CO_2_H (**8**) and NO_2_ (**9**), respectively (Figure 2). 

**Figure 3:**
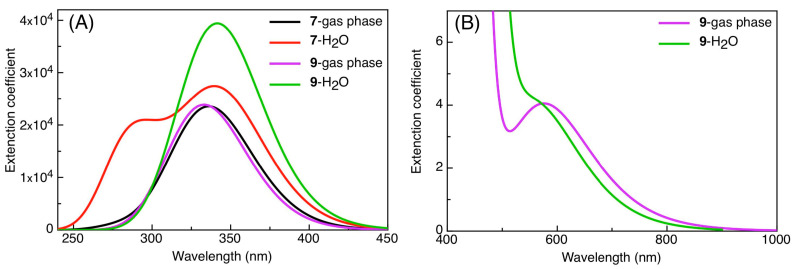
(**A**) Simulated absorption spectra of compounds **7** and **9** calculated by TD-DFT in the gas phase and H_2_O, (**B**) Simulated absorption spectra of 9 in the gas phase and H_2_O at longer wavelength

The biological activities of molecules, such as antitumor, anticancer, and antioxidant activities, depend on the facile electron/proton transfer process. For example, the ease of the oxidation process largely depends on electron excitation and transition within biological active molecules. Hence, simulated UV-Vis spectra in the gas phase and in H_2_O were analyzed. In order to simulate the absorption spectrum of (**4**–**9**) derivatives, time-dependent density functional theory (TD-DFT) calculations based on the optimized structures at the B3LYP/6-311++G(d,p) level of theory were performed individually in the gas phase and in aqueous medium [28]. The calculated HOMO and LUMO orbitals of the triazole derivatives (**4**–**9**), are presented in Table 1, while the calculated HOMO and LUMO energies and the corresponding energy gap are shown in Table 2. An analysis of the data revealed that both the HOMO and LUMO orbitals are situated on pyrazole moieties in **5**, **7**, and **9** analogies, whereas the substituted phenyl moiety participates in the LUMO orbitals in **4**, **6**, and **8** derivatives. The HOMO orbitals’ energies for compounds **4–9** are similar in the gas phase and in aqueous medium, with analogies **6** and **7** showing the lowest HOMO energy values. In addition, the calculated energy gap (E_g_) was slightly changed by the electron donor groups, as demonstrated in **4**, **5**, **6**, and **7** triazole derivatives, where a significant decrease in the energy gap were observed for electron-pulling substituents such as compound **8** (CO_2_H) and **9** (NO_2_) derivatives [28,29,30]. The low HOMO-LUMO energy gap values show that the triazole derivatives are biologically active agents. The calculated absorption spectral wavelengths (*λ*_max_) and corresponding oscillator strengths are shown in Table 2. The simulated absorption spectra of the **7** and **9** derivatives can be seen in Figure 3A in the gas phase and in aqueous medium. Phenyl substituents slightly affected the electron transition in (**4**–**8)** derivatives. Compared with unsubstituted phenyl group (**4**), the electron-donating group increased the absorption spectra wavelength (Bathochromic), while the electron-withdrawing groups such as CO_2_H (**8**) and NO_2_ (**9**) lowered the absorption spectra wavelength (Hypsochromic) (Table 2). Interestingly, the NO_2_-phenyl-substituted derivative shows an extra absorption peak at 580 nm (Figure 3B) [28], which shows a hypsochromic shift in aqueous medium. On the other hand, derivative 7 (-NH2) shows a low energy absorption peak at 291 nm in aqueous medium, which is ascribed to the solvent–solute interaction.

### 2.3. Pharmacological Evaluation

#### 2.3.1. Cytotoxic Impacts

**Figure 4:**
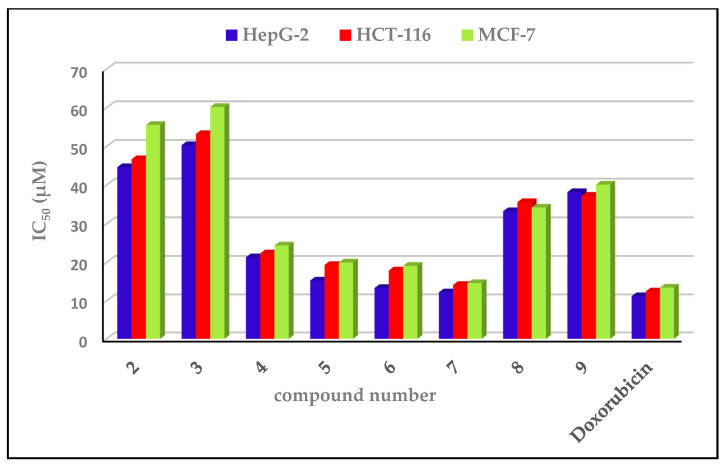
*In vitro* anticancer effects of the target compounds on the HepG-2, HCT-116, and MCF-7 cell lines.

The cytotoxicity of a series of eight representative conjugates was assessed *in vitro* against three human tumor cell lines—a human colon cancer cell line (HCT-116), human breast cancer cell line (MCF-7), and human liver cancer cell line (HepG2)—following the Sulforhodamine B protocol [31,32]. The activity of the screened compounds was compared to that of the doxorubicin drug (positive control), while DMSO was the negative control. IC_50_ was the parameter used which corresponds to the required concentration for a 50% cell viability inhibition. The outcomes of cytotoxicity are offered in Table 3 and Figure 4.

An analysis of the obtained data revealed that all the target compounds (**2**–**9)** recorded good activity against the HepG-2, HCT-116, and MCF-7 cell lines (IC_50_ = 12.22–50.34, 14.16–53.21, and 14.64–60.20 µM, respectively), compared with doxorubicin as a standard (IC_50_ = 11.21, 12.46 and 13.45 µM, respectively). Compound **7** was the most potent candidate towards HepG-2, HCT-116, and MCF-7, with an IC_50_ = 12.22, 14.16, and 14.64 µM, respectively. Furthermore, compound **7** recorded a comparable cytotoxic potential (IC_50_ = 12.22, 14.16, and 14.64 µM) to that exhibited by doxorubicin (IC_50_ = 11.21, 12.46, and 13.45 µM). The pyrazolo-[1,2,4]triazole derivatives **2** (IC_50_ = 44.65, 46.71, and 55.57 µM, respectively) and **3** (IC_50_ = 50.34, 53.21, and 60.20 µM, respectively) exhibited the least potency towards the three cell lines, HepG-2, HCT-116, and MCF-7. Within 4-substitutedphenyl-[1,2,3]-triazole derivatives (**4**–**9**), for the three cell lines (HepG-2, HCT-116, and MCF-7) the 4-aminophenyl derivative **7** showed a higher potency (IC_50_ = 12.22, 14.16, and 14.64 µM, respectively) > 4-methoyphenyl derivative **6** (IC_50_ = 13.36, 17.93, and 19.14 µM, respectively) > 4-methylphenyl derivative **5** (IC_50_ = 15.31, 19.35, and 20.00 µM, respectively) > unsubstituted-phenyl derivative **4** (IC_50_ = 21.35, 22.35, and 24.41 µM, respectively) > 4-carboxy derivative **8** (IC_50_ = 33.26, 35.61, and 34.21 µM, respectively) > 4-nitrophenyl derivative **9** (IC_50_ = 38.20, 37.24, and 40.14 µM, respectively). 

#### 2.3.2. Structure–Activity Relationship

The analysis of the cytotoxic impacts revealed that there was some structure–activity relationship; for example, adding the [1,2,3]-triazole moiety to the pyrazolo-[1,2,4]-triazole scaffold markedly favored the cytotoxic potential against the HepG-2, HCT-116, and MCF-7 cell lines. This is obvious upon comparing pyrazolo-[1,2,4]-triazole derivatives **2** (IC_50_ = 44.65, 46.71, and 55.57 µM, respectively) and **3** (IC_50_ = 50.34, 53.21, and 60.20 µM, respectively) with the pyrazole-[1,2,4]-triazole-[1,2,3]-triazole hybrids (**4**–**9**) (IC_50_ = 12.22–38.20, 14.16–37.24, and 14.64–40.14 µM, respectively). It noteworthy that the analogies with electron-releasing substitutions in the phenyl ring attached to N-1 of 1,2,3-triazole demonstrated a higher cytotoxic potential against all tested tumor lines than those with electron-pulling substituents, as shown in compounds **5** (IC_50_ = 15.31, 19.35, and 20.00 µM, respectively), **6** (IC_50_ = 13.36, 17.93, and 19.14, µM, respectively), and **7** (IC_50_ = 12.22, 14.16, and 14.64 µM), compared with derivatives **8** (IC_50_ = 33.26, 35.61, and 34.21 µM, respectively) and **9** (IC_50_ = 38.20, 37.24, and 40.14 µM, respectively). 

### 2.4. Docking Study

**Figure 5:**
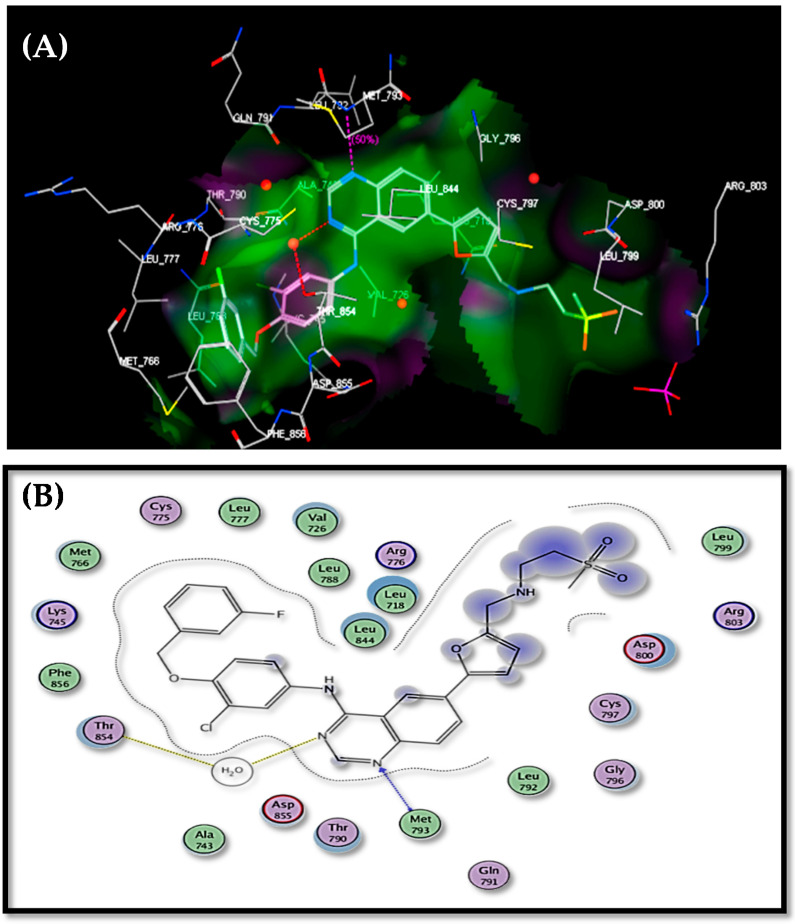
Docking of lapatinib within the ATP binding site of EGFR: (**A**) 3D binding mode; (**B**) 2D binding mode.

Epidermal growth factor receptor (EGFR) constitutes a family of trans-membrane growth factor protein tyrosine kinases [33,34]. The EGFR family has a major role in cell survival and signal transduction [35]. The overexpression of EGFR has been implicated in many tumors of epithelial origin (breast, colon, ovarian, and NSC lung cancer) [36]. Thus, targeting EGFR represents a strategy for the design of selective anticancer agents [37]. To detect the mode of anticancer potential of the targets candidates (**2**–**9)**, we docked these candidates into the ATP binding region of epidermal growth factor receptor (EGFR) using MOE, 2010, Version 8. The X-ray crystal of EGFR cocrystallized with Lapatinib was downloaded from the protein data bank (PDB: 1XKK) [38,39]. Lapatinib was redocked within the active site with a root mean standard deviation (RMSD) = 1.6452. Lapatinib recorded a score energy = −17.22 B3LYP. N-3 of quinazoline ring formed a H_2_O-mediated H bond to the side chain of Thr854, and N-1 was hydrogen bonded to Met793 (Figure 5). 

**Figure 6:**
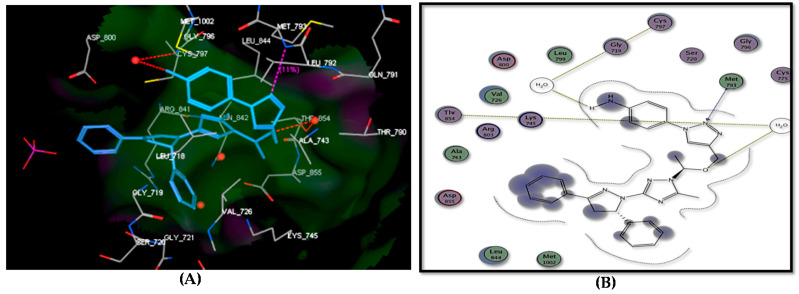
Docking of compound **7** within the ATP binding site of EGFR: (**A**) 3D binding mode; (**B**) 2D binding mode.

The outcomes from docking the target compounds inside the EGFR binding site as binding energy scores and hydrogen bonding are presented in Table 4. An analysis of these results revealed that congener **7** recorded the best docking score (−17.01 kcal/mol) and performed the same binding mode inside the EGFR active site as lapatinib. It formed three H bonds with Thr854 (*via* H_2_O), Met793, and Cys797 through binding with OCH_2_ and N-2 of the triazole and amino groups (Figure 6). Furthermore, the candidate **6** revealed a docking score = −16.70 kcal/mol and formed two hydrogen bonds with Thr854 (*via* H_2_O) and Lys745 through binding with Me-O and N-2 of 1,2,3-triazole (Figure S3). Regarding compound **5**, it displayed a good fitting inside the active site, forming an arene–cation interaction with Arg841 in addition to the formation of two H-bonds in the following way: (i) Thr854 (*via* H_2_O) with N-2 of triazole; (ii) Lys745 with OCH_2_ (Figure S4). The unsubstituted phenyl analogy **4** achieved a binding energy score of −16.22 kcal/mol and formed one hydrogen bond with Thr854 *via* binding with N-2 of the triazole moiety (Figure S5). Meanwhile, compound **8** showed a binding score of −15.02 kcal/mol and formed H bonds with Thr854 and Cys797 amino acids (Figure S6). Compound **9** revealed one hydrogen bond with Thr854 *via* binding with N-2 of the pyrazole ring with a binding score equal to −14.67 kcal/mol (Figure S7).

Finally, target compounds **2** and **3** recorded the least binding scores of −14.50 kcal/mol (with compound **2**) and −14.34 (with compound **3**). Furthermore, candidates **2** and **3** formed only one hydrogen bond within the active site. In the case of compound **2**, the hydroxy group of this derivative hydrogen bound with Met793 (Figure S8), while N-2 of the pyrazole moiety in compound **3** hydrogen-bound with Cys797 amino acid (Figure S9).

In brief, all the docked compounds recorded a good fitting inside the EGFR active site with a binding energy score in the range of −17.01 to −14.34 kcal/mol compared to the cocrystallized ligand lapatinib (binding energy score = −7.22 kcal/mol). In addition, all the docked derivatives formed 1 to 3 hydrogen bonds within the active site through binding with Met793, Thr854, Lys745, and/or Cys797 amino acids. Furthermore, compound **7** (the most potent candidate towards HepG-2, HCT-116, and MCF-7) displayed the best docking score (−17.01 kcal/mol) and showed same binding mode as lapatinib. While, compounds **2** and **3** (the least potent derivatives towards the tested cell lines) revealed the lowest binding scores of −14.50 kcal/mol and −14.34, respectively, and formed only one hydrogen bond within the active site. These results were in agreement with those obtained from the *in vitro* cytotoxic assay.

## 3. Materials and Methods

### 3.1. General Description of Materials and Methods

All the chemicals and solvents were bought from Sigma Aldrich (Bayouni Trading Co. Ltd., Taif, KSA). Reactions were run at room temperature (20–25 °C), unless otherwise noted in the experimental procedure, and reported reaction temperatures refer to the external temperatures measured for the bath in which the reaction vessel was immersed. Heating was obtained through the use of a silicone oil bath. The removal of residual solvents was accomplished by the evacuation of the container for a period of 12–20 hrs using a high vacuum line. The reaction progress was controlled by thin-layer chromatography studies (TLC) on pre-coated silica gel 60-F_254_ on aluminum sheets (Merck, Darmstadt, Germany). The spots were detected and visualized by UV illumination (254 nm). 

### 3.2. Instrumentation

The structures of all the synthesized compounds were confirmed with ^1^H-NMR, ^13^C-NMR, IR, elemental, and MS analyses. The melting points were measured by a numerical Gallen-Kamp MFB-595 apparatus (Gallenkamp, London, UK). IR spectra (KBr) were verified on a Bruker-Vector 22 FTIR spectrophotometer (Bruker, Manasquan, NJ, USA). A Varian Mercury VXR-300 (Bruker, Marietta, GA, USA) at 300 and 75 MHz for the ^1^H and ^13^C-NMR spectra, respectively, in DMSO-*d_6_* was used to collect the NMR spectra. Chemical shifts are expressed in ppm with respect to the residual solvent signal. Mass spectra were measured at 70 eV on a Hewlett Packard MS-5988 spectrometer (Hewlett Packard, Palo Alto, CA, USA). The elemental analyses were performed at the Micro-Analytical Center of Taif University, Taif, KSA. 

### 3.3. Synthetic Procedures and Analytic Data of Compounds 

1-(3-(3,5-Diphenyl-4,5-dihydro-1*H*-pyrazol-1-yl)-5-methyl-1*H*-1,2,4-triazol-1-yl) ethanol (**2**). To a solution of acetyl 1,2,4-triazole **1** [26] (0.345g, 0.1 mol) in dry MeOH (30 mL), a solution of NaBH_4_ (0.1 g) in dry MeOH (10 mL) was added dropwise and the reaction mixture was stirred at RT for 1 h. (TLC monitored). Afterwards, the solvent was evaporated under vacuum and the residue was triturated with ethyl acetate to yield the primary alcohol analogy **2** as a faint yellow amorphous mass in an 85% yield; m.p. 249–251 °C; IR (KBr, ν_max_, cm^−1^): 1612-1623 (3C=N), 2858 (Me/CH_2*sym*_), 2923 (Me/CH_2 *asym*_), 3380 (O-H); ^1^H-NMR δ: 2.35, 2.49 (s, 6H, 2Me), 3.54 (dd, *J* = 12.6, 4.8 Hz, 1H), 3.67 (s, 1H, OH _Deutr. Exch_), 3.95 (dd, *J* = 8.8, 13.2 Hz, 1H), 5.18 (dd, *J* = 10.1, 4.1 Hz, 1H), 5.61 (s, 1H, -CH-OH), 7.16–7.75 (m, 10H, Ar–H); ^13^C-NMR δ: 11.0, 20.1 (2 Me), 40.1, 55.4 (2CH_2_), 59.7, 83.7 (2CH), 126.5, 126.8, 128.0, 128.4, 128.5, 134.4, 137.6, 143.8 (C–Ar), 147.0, 151.4, 152.7 (3 C=N–); MS (*m/z*, %): 347.17 (M^+^, 18). Anal. Calcd for C_20_H_21_N_5_O (347.41): C, 69.14; H, 6.09; N, 20.16%. Found: C, 69.02; H, 6.00; N, 20.01%. 

3-(3,5-Diphenyl-4,5-dihydro-1*H*-pyrazol-1-yl)-5-methyl-1-(1-(prop-2-yn-1-yloxy)ethyl)-1*H*-1,2,4-triazole (**3**). To a solution of hydroxymethyl compound **2** (0.347 g, 0.1 mol) along with K_2_CO_3_ (0.20 g, 1.5 mmol) in DMF (20 mL), a solution of propargyl bromide (1.5 mol) in DMF (10 mL) was added. Afterwards, the reaction mixture was refluxed under stirring for 6 h (checked by TLC). The solvent was partially evaporated and the formed solid was collected and recrystallized using EtOH to furnish the propargyl analogy **3** as a red amorphous mass in an 75% yield; m.p. 143–145 °C; IR (KBr, ν_max_, cm^−1^): 1063 (C−O−C), 1612-1623 (3C=N), 2120 (C≡C), 2858 (Me/CH_2*sym*_), 2923 (Me/CH_2 *asym*_), 3257 (≡C−H); ^1^H-NMR δ: 2.35, 2.49 (s, 6H, 2Me), 3.20 (s, 1H, ≡C−H), 3.54 (dd, *J* = 12.6, 4.8 Hz, 1H), 3.95 (dd, *J* = 8.8, 13.2 Hz, 1H), 4.10 (s, 2H, O-CH_2_), 5.18 (dd, *J* = 10.1, 4.1 Hz, 1H), 5.61 (s, 1H, -CH-O-), 7.16–7.75 (m, 10H, Ar–H); ^13^C-NMR δ: 11.0, 17.0 (2 Me), 40.1, 55.4 (CH_2_), 59.7, 89.7 (2CH), 76.4, 79.0 (C≡C), 126.5, 126.8, 128.0, 128.4, 128.5, 134.4, 137.6, 143.8 (C–Ar), 147.0, 151.4, 152.7 (3C=N–); MS (*m/z*, %): 385.19 (M^+^, 26). Anal. Calcd for C_23_H_23_N_5_O (385.46): C, 71.67; H, 6.01; N, 18.17%. Found: C, 71.39; H, 6.04; N, 18.01%. 

Synthesis of 1-(Substituted phenyl)-4-((1-(3-(3,5-diphenyl-4,5-dihydro-1*H*-pyra- zol-1-yl)-5-methyl-1*H*-1,2,4-triazol-1-yl)ethoxy)methyl)-1*H*-1,2,3-triazoles (**4**–**9**). A mixture of propargyl substrate 3 (0.385 g, 10.1 mol), aryl azide derivatives (0.1 mol), Na-ascorbate (0.1 mol), and CuSO_4_·5H_2_O (0.25 mmol) in DMF−H_2_O (6:1, 8 mL) was stirred at 60–70 °C for 5–7 h (TLC monitored). Afterwards, the reaction mixture was quenched in H_2_O (100 mL) and extracted thrice with ethyl acetate (20 mL). The raw matter was purified by flash chromatography (toluene: ethyl acetate (7:3)), to furnish the target *N*-(substituted phenyl)-1,2,3-triazole analogies (**4**–**9**).

4-((1-(3-(3,5-Diphenyl-4,5-dihydro-1*H*-pyrazol-1-yl)-5-methyl-1*H*-1,2,4-triazol-1-yl)ethoxy)methyl)-1-phenyl-1*H*-1,2,3-triazole (**4**). Yield (75%) from toluene: ethyl acetate, 7:3, as a yellow powder; *R*f 0.32 (toluene: ethyl acetate (7:3)); m.p. 213–215 °C; IR (KBr, ν_max_, cm^−1^): 1067 (C−O−C), 1610-1623 (3C=N), 2858 (Me/CH_2*sym*_), 2923 (Me/CH_2 *asym*_); ^1^H-NMR δ: 2.35, 2.49 (s, 6H, 2Me), 3.54 (dd, *J* = 12.6, 4.8 Hz, 1H), 3.95 (dd, *J* = 8.8, 13.2 Hz, 1H), 4.60 (s, 2H, O-CH_2_), 5.18 (dd, *J* = 10.1, 4.1 Hz, 1H), 5.35 (s, 1H, -CH-O-), 7.16–7.75 (m, 15H, Ar–H), 8.21 (s, 1H, Triazole._(C4)_-H); ^13^C-NMR δ: 11.0, 19.0 (2Me), 40.1, 64.4 (CH_2_), 59.7, 88.9 (2CH), 119.1, 119.6, 125.6, 125.8, 128.1, 128.4, 129.1, 133.7, 137.6, 143.8, 144.4 (C–Ar), 148.6, 151.7, 153.3 (3 C=N–); MS (*m/z*, %): 504.24 (M^+^, 34). Anal. Calcd for C_29_H_28_N_8_O (504.59): C, 69.03; H, 5.59; N, 22.21%. Found: C, 68.93; H, 5.23; N, 22.01%. 

4-((1-(3-(3,5-Diphenyl-4,5-dihydro-1*H*-pyrazol-1-yl)-5-methyl-1*H*-1,2,4-triazol-1-yl)ethoxy)methyl)-1-(p-tolyl)-1*H*-1,2,3-triazole (**5**). Yield (76%) from toluene: ethyl acetate, 7:3, as a yellow amorphous mass; *R*f 0.42 (toluene: ethyl acetate (7:3)); m.p. 223–225 °C; IR (KBr, ν_max_, cm^−1^): 1067 (C−O−C), 1610-1623 (3C=N), 2858 (Me/CH_2*sym*_), 2923 (Me/CH_2 *asym*_); ^1^H-NMR δ: 2.35–2.49 (s, 9H, 3Me), 3.54 (dd, *J* = 12.6, 4.8 Hz, 1H), 3.95 (dd, *J* = 8.8, 13.2 Hz, 1H), 4.59 (s, 2H, O-CH_2_), 5.18 (dd, *J* = 10.1, 4.1 Hz, 1H), 5.31 (s, 1H, -CH-O-), 7.16–7.75 (m, 14H, Ar–H), 8.21 (s, 1H, Triazole._(C4)_-H); ^13^C-NMR δ: 11.0, 19.0, 21,1 (3Me), 40.1, 64.4 (CH_2_), 59.7, 88.9 (2CH), 119.1, 119.6, 125.6, 125.8, 128.1, 128.4, 129.1, 133.7, 137.6, 143.8, 144.4 (C–Ar), 148.6, 151.7, 153.3 (3 C=N–); MS (*m/z*, %): 519.25 (M^+^+ 1, 4). Anal. Calcd for C_30_H_30_N_8_O (518.61): C, 69.48; H, 5.83; N, 21.61%. Found: C, 69.21; H, 5.49; N, 21.37%.

4-((1-(3-(3,5-Diphenyl-4,5-dihydro-1*H*-pyrazol-1-yl)-5-methyl-1*H*-1,2,4-triazol-1-yl)ethoxy)methyl)-1-(4-methoxyphenyl)-1*H*-1,2,3-triazole (**6**). Yield (83%) from toluene: ethyl acetate, 7:3, as a faint yellow amorphous mass; *R*f 0.25 (toluene: ethyl acetate (7:3)); m.p. 193–195 °C; IR (KBr, ν_max_, cm^−1^): 1051-1076 (2C−O−C), 1610-1623 (3C=N), 2858 (Me/CH_2 *sym*_), 2923 (Me/CH_2 *asym*_); ^1^H-NMR δ: 2.35, 2.49 (s, 6H, 2Me), 3.54 (dd, *J* = 12.6, 4.8 Hz, 1H), 3.64 (s, 3H, O-Me), 3.95 (dd, *J* = 8.8, 13.2 Hz, 1H), 4.61 (s, 2H, O-CH_2_), 5.18 (dd, *J* = 10.1, 4.1 Hz, 1H), 5.35 (s, 1H, -CH-O-), 7.16–7.75 (m, 14H, Ar–H), 8.11 (s, 1H, Triazole._(C4)_-H); ^13^C-NMR δ: 11.0, 19.0 (2Me), 40.1, 64.4 (CH_2_), 55,8 (O-Me), 59.7, 88.9 (2CH), 119.1, 119.6, 125.6, 125.8, 128.1, 128.4, 129.1, 133.7, 137.6, 143.8, 144.4 (C–Ar), 148.6, 151.7, 153.3 (3 C=N–); MS (*m/z*, %): 534.25 (M^+^, 25). Anal. Calcd for C_30_H_30_N_8_O_2_ (534.61): C, 67.40; H, 5.66; N, 20.96%. Found: C, 67.21; H, 5.34; N, 20.69%.

4-(4-((1-(3-(3,5-Diphenyl-4,5-dihydro-1*H*-pyrazol-1-yl)-5-methyl-1*H*-1,2,4-triazol-1-yl)ethoxy)methyl)-1*H*-1,2,3-triazol-1-yl)aniline (**7**). Yield (80%) from toluene: ethyl acetate, 7:3, as a red amorphous mass; *R*f 0.30 (toluene: ethyl acetate (7:3)); m.p. 243–245 °C; IR (KBr, ν_max_, cm^−1^): 1067 (C−O−C), 1610-1623 (3C=N), 2858 (Me/CH_2 *sym*_), 2923 (Me/CH_2 *asym*_), 3315 (N-H_2_); ^1^H-NMR δ: 2.35, 2.49 (s, 6H, 2Me), 3.54 (dd, *J* = 12.6, 4.8 Hz, 1H), 3.95 (dd, *J* = 8.8, 13.2 Hz, 1H), 4.61 (s, 2H, O-CH_2_), 5.18 (dd, *J* = 10.1, 4.1 Hz, 1H), 5.35 (s, 1H, -CH-O-), 6.39 (s, 2H, N-H_2 Deutr. Exch_), 7.16–7.75 (m, 14H, Ar–H), 8.09 (s, 1H, Triazole._(C4)_-H); ^13^C-NMR δ: 11.0, 19.0 (2Me), 40.1, 64.4 (CH_2_), 60.2, 90.5 (2CH), 119.1, 124.2, 126.7, 126.8, 126.9, 128.2, 128.5, 128.8, 130.1, 131.0, 136.4, 143.5, 144.4, 148.4 (C–Ar), 148.8, 151.6, 152.3 (3 C=N–); MS (*m/z*, %): 519.25 (M^+^, 5). Anal. Calcd for C_29_H_29_N_9_O (519.60): C, 67.03; H, 5.63; N, 24.26%. Found: C, 66.89; H, 5.38; N, 24.05%.

4-(4-((1-(3-(3,5-Diphenyl-4,5-dihydro-1*H*-pyrazol-1-yl)-5-methyl-1*H*-1,2,4-triazol-1-yl)ethoxy)methyl)-1*H*-1,2,3-triazol-1-yl)benzoic acid (**8**). Yield (76%) from toluene: ethyl acetate, 7:3, as a reddish-brown amorphous mass; Rf 0.21 (toluene: ethyl acetate (7:3)); m.p. 265–267 °C; IR (KBr, ν_max_, cm^−1^): 1067 (C−O−C), 1610-1623 (3C=N), 2858 (Me/CH_2 *sym*_), 2923 (Me/CH_2 *asym*_), 3485 (CO_2_H); ^1^H-NMR δ: 2.35, 2.49 (s, 6H, 2Me), 3.54 (dd, *J* = 12.6, 4.8 Hz, 1H), 3.95 (dd, *J* = 8.8, 13.2 Hz, 1H), 4.61 (s, 2H, O-CH_2_), 5.18 (dd, *J* = 10.1, 4.1 Hz, 1H), 5.35 (s, 1H, -CH-O-), 6.86–7.75 (m, 14H, Ar–H), 8.09 (s, 1H, Triazole._(C4)_-H), 10.75 (s, 1H, O-H); ^13^C-NMR δ: 11.0, 19.0 (2Me), 40.1, 64.4 (CH_2_), 59.7, 88.9 (2CH), 119.1, 119.6, 125.6, 125.8, 128.1, 128.4, 129.1, 133.7, 137.6, 143.8, 144.4 (C–Ar), 148.6, 151.7, 153.3 (3 C=N–), 164.4 (C=O); MS (*m/z*, %): 548.23 (M^+^, 14). Anal. Calcd for C_30_H_28_N_8_O_3_ (548.60): C, 65.68; H, 5.14; N, 20.43%. Found: C, 65.39; H, 5.01; N, 20.21%.

4-((1-(3-(3,5-Diphenyl-4,5-dihydro-1*H*-pyrazol-1-yl)-5-methyl-1*H*-1,2,4-triazol-1-yl)ethoxy)methyl)-1-(4-nitrophenyl)-1*H*-1,2,3-triazole (**9**). Yield (85%) from toluene: ethyl acetate, 7:3, as a red amorphous mass; Rf 0.25 (toluene: ethyl acetate (7:3)); m.p. 179–181 °C; IR (KBr, ν_max_, cm^−1^): 1067 (C−O−C), 1395 (NO_2_*_sym_*), 1520 (NO_2_*_asym_*), 1610-1623 (3C=N), 2858 (Me/CH_2 *sym*_), 2923 (Me/CH_2 *asym*_); ^1^H-NMR δ: 2.35, 2.49 (s, 6H, 2Me), 3.54 (dd, *J* = 12.6, 4.8 Hz, 1H), 3.95 (dd, *J* = 8.8, 13.2 Hz, 1H), 4.61 (s, 2H, O-CH_2_), 5.18 (dd, *J* = 10.1, 4.1 Hz, 1H), 5.35 (s, 1H, -CH-O-), 7.16–7.75 (m, 14H, Ar–H), 8.19 (s, 1H, Triazole._(C4)_-H); ^13^C-NMR δ: 11.0, 19.0 (2Me), 40.1, 64.4 (CH_2_), 59.7, 88.9 (2CH), 119.1, 119.6, 125.6, 125.8, 128.1, 128.4, 129.1, 133.7, 137.6, 143.8, 144.4 (C–Ar), 148.6, 151.7, 153.3 (3 C=N–); MS (*m/z*, %): 549.22 (M^+^, 42). Anal. Calcd for C_29_H_27_N_9_O_3_ (549.58): C, 63.38; H, 4.95; N, 22.94%. Found: C, 63.17; H, 4.63; N, 22.70%.

### 3.4. Cytotoxic Assessment

#### Methodology

The preliminary *in vitro* cytotoxic assay against a human breast cancer cell line (MCF-7), human colon cancer cell line (HCT-116), and human liver cancer cell line (HepG2) was achieved according to the SRB method as previously reported [31,32].

### 3.5. Molecular Docking Study

The crystal structure of epidermal growth factor receptor (EGFR) cocrystallized with Lapatinib was downloaded from protein data bank (PDB file: 1XKK) [35]. The MOE 2010 program was used for carrying out molecular docking for the target derivatives **2**–**9** inside the EGFR active site. The cocrystallized ligand was redocked inside the dynamic site in order to ensure the veracity of the docking study and the RMSD was determined. The 3D structures of the prepared compounds were built by the MOE molecular builder, then protonated, followed by energy minimization, then saved in an mdb file to be docked inside the active site of EGFR. Hydrogen bonds, interacting groups, and docking scores are listed in Table 2.

### 3.6. DFT Details

The DFT and TD-DFT methods in the Gaussian 09 program [40] were used to investigate the structures’ geometries. The structures were optimized using the B3LYP level and the 6-311++G(d,p) basis set. We performed frequency calculations in order to confirm the structures’ stability. The absorption spectra were simulated by TD-DFT calculations. We used the self-consistent reaction field (SCRF) and polarizable continuum model (PCM) for the solvent effect calculations.

## 4. Conclusions

In conclusion, we have reported the synthesis and *in vitro* cytotoxic assay of novel 1,2,3-triazole-contanning hybrids. These frameworks were easily achieved in an excellent yield by click chemistry through a copper(I)-catalyzed azide alkyne cycloaddition reaction (CuAAC). The DFT calculations demonstrated that phenyl substituent modulated the photophysical properties of (**4**–**9**) derivatives. In addition, electron-releasing substituents increased the absorption spectra (bathochromic shift), whereas electron-pulling substituents decreased the absorption wavelength (hypsochromic shift). The *in vitro* cytotoxic assay disclosed that, among the tested 1,2,3-triazole derivatives, the congeners **7**, **6**, and **5** exhibited momentous cytotoxicity, with IC_50_ of 12.22, 13.36, and 15.3 μM, respectively. Concerning the 1,2,3-triazole derivatives, derivative **7** was the most potent candidate towards HepG-2, HCT-116, and MCF-7, with an IC_50_ = 12.22, 14.16, and 14.64 µM, respectively, in comparison to the effect exhibited by doxorubicin (IC_50_ = 11.21, 12.46, and 13.45 µM). Moreover, a SAR study revealed that substituting the phenyl ring with electron-releasing substituents exhibited a higher cytotoxic impact towards the three cell lines than using an electron-withdrawing group, as demonstrated by congeners **5** (IC_50_ = 15.31, 19.35, and 20.00 µM, respectively), **6** (IC_50_ = 13.36, 17.93, and 19.14, µM, respectively), and **7** (IC_50_ = 12.22, 14.16, and 14.64 µM), compared with derivatives **8** (IC_50_ = 33.26, 35.61, and 34.21 µM, respectively) and **9** (IC_50_ = 38.20, 37.24, and 40.14 µM, respectively). Moreover, a docking study was carried out on the target compounds **2**–**9** within the active site of EGFR to predict the mechanism of effect of these targets. This study revealed a good fitting for candidates **2**–**9** within the EGFR active site.

## Figures and Tables

**Table 1:** Calculated HOMO and LUMO orbitals for compounds **4**–**9** by (B3LYP/6-311++G(d,p)) level of theory in the gas phase.

Comp. No	HOMO	LUMO
4	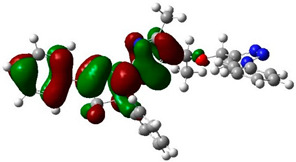	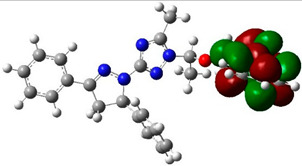
5	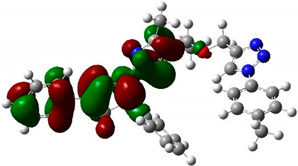	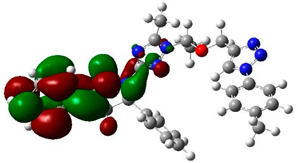
6	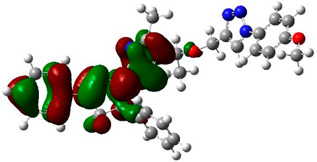	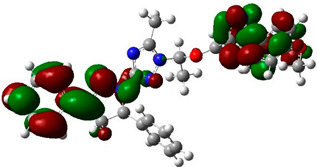
7	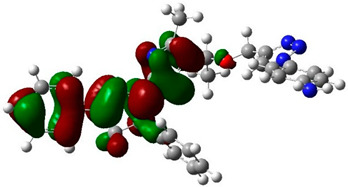	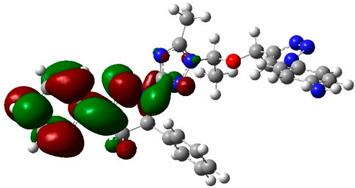
8	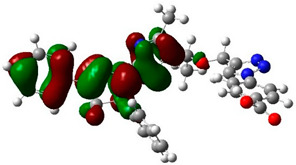	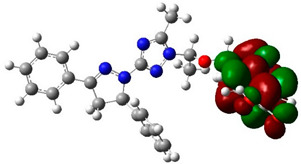
9	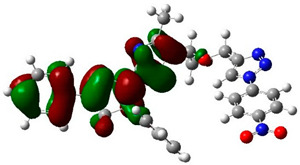	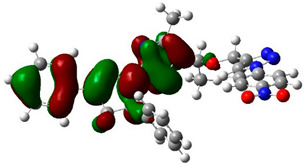

**Table 2:** HOMO orbital energies (E_HOMO_), LUMO orbital energies (E_LUMO_), energy gap (E_g_), oscillator strength (*f*) calculated by the TD-DFT in gas phase and H_2_O.

Comp.	E_HOMO_ (eV)	E_LUMO_ (eV)	E_g_ (eV)	*λ*_max_ (nm)	*f*
4	−5.43 (−5.68) ^a^	−1.42 (−1.61)	4.01 (4.07)	334 (341)	0.58
5	−5.42 (−5.69)	−1.35 (−1.62)	4.07 (4.07)	336 (341)	0.58
6	−5.30 (−5.66)	−1.26 (−1.60)	4.04 (4.06)	340 (343)	0.59
7	−5.39 (−5.67)	−1.32 (−1.61)	4.07 (4.06)	337 (291, 341)	0.58
8	−5.54 (−5.68)	−2.34 (−2.26)	3.20 (3.42)	333 (341)	0.59
9	−5.55 (−5.68)	−3.13 (−3.21)	2.42 (2.47)	333, 580 (343, 585)	0.58

^a^ The data in brackets in H_2_O.

**Table 3:** Preliminary *in vitro* cytotoxicity values of the target derivatives **2–9** and doxorubicin.

Compound No.	IC_50_ (µM) ± SD		
	HepG-2	HCT-116	MCF-7
2	44.65 ± 2.03	46.71 ± 1.35	55.57 ± 1.89
3	50.34 ± 1.18	53.21 ± 0.31	60.20 ± 0.36
4	21.35 ± 0.67	22.35 ± 0.96	24.41 ± 0.36
5	15.31 ± 0.65	19.35 ± 1.41	20.00 ± 0.46
6	13.36 ± 0.24	17.93 ± 0.53	19.14± 0.87
7	12.22 ± 1.02	14.16 ± 0.38	14.64 ± 0.64
8	33.26 ± 0.62	35.61 ± 1.21	34.21 ± 1.01
9	38.20 ± 1.61	37.24 ± 0.32	40.14 ± 1.05
Doxorubicin	11.21± 0.14	12.46 ± 0.19	13.45 ± 0.54

**Table 4:** Docking data for compounds **2**–**9** within EGFR enzyme (PDB: 1XKK).

Target No.	E. Score Kcal/mol	hydrogen Bonds Number	Distance (A^o^) from Amino Acid		Binded Group
**2**	−14.50	1	3.05	Met793	OH
**3**	−14.34	1	2.98	Cys797	N-2 of pyrazole
**4**	−16.22	1	2.82	Thr854	N-2 of triazole
**5**	−15.83	2	3.54 2.75	Thr854 (*via* H_2_O) Lys745	N-2 of triazole OCH_2_
**6**	−16.70	2	2.83 2.59	Thr854 (*via* H_2_O) Lys745	OMe N-2 of triazole
**7**	−17.01	3	2.44 2.48 2.97	Thr854 (*via* H_2_O) Met793 Cys797	OCH_2_ N-2 of triazole NH_2_
**8**	−15.02	2	3.22 2.98	Cys797 (*via* H_2_O) Thr854 (*via* H_2_O)	N-3 of triazole CO_2_H
**9**	−14.67	1	2.79	Thr854	N-2 of pyrazole
Lapatinib	−17.22	2	2.30 2.41	Thr854 (*via* H_2_O) Met793	N-3 of quinazoline N-1 of quinazoline

## Data Availability

The data presented in this study are available in this article.

## Supplementary Materials

The following are available online: Figure S1: Optimized structure of **4** and **5** derivatives at B3LYP /6-311++G(d,p) level of theory in the gas phase, Figure S2: Optimized structure of **6** and **7** derivatives at B3LYP/6-311++G(d,p) level of theory in the gas phase, Figure S3. Docking of compound 6 within ATP bind site of EGFR. (**A**) 3D biding mode. (**B**) 2D binding mode. Figure S4. Docking of compound **5** within ATP bind site of EGFR. (**A**) 3D biding mode. (**B**) 2D binding mode. Figure S5. Docking of compound **4** within ATP bind site of EGFR. (**A**) 3D biding mode. (**B**) 2D binding mode. Figure S6. Docking of compound **8** within ATP bind site of EGFR. (**A**) 3D biding mode. (**B**) 2D binding mode. Figure S7. Docking of compound **9** within ATP bind site of EGFR. (**A**) 3D biding mode. (**B**) 2D binding mode. Figure S8. Docking of compound **2** within ATP bind site of EGFR. (**A**) 3D biding mode. (**B**) 2D binding mode. Figure S9. Docking of compound **3** within ATP bind site of EGFR. (**A**) 3D biding mode. (**B**) 2D binding mode.

## Sample Availability

Samples of compounds **2**–**9** are available from the authors.
