# Supplementary material for: New 1,2,3-Triazole-Containing Hybrids as Antitumor Candidates: Design, Click Reaction Synthesis, DFT Calculations, and Molecular Docking Study
Source: Molecules. 2021 Jan 29;26(3):708. doi: 10.3390/molecules26030708 (PMC7866392; doi:10.3390/molecules26030708)
Supplement: Supplementary file 1 [file molecules-26-00708-s001.pdf]

Supplementary Data

New 1,2,3-triazole-containing hybrids as antitumor candidates: Design, click-reaction synthesis, DFT calculations and molecular docking study

Islam H. El Azab[1,2,*] , Hamdy S. El-Sheshtawy[3], Rania B. Bakr[4,5] and Nadia A. A. Elkanzi[2,6]

[1] Chemistry Department, College of Science, Taif University, P.O. box 11099, Taif 21944, Saudi Arabia.
[2] On leave from Chemistry Department, Faculty of Science, Aswan University, Aswan, P.O. box 81528, Aswan, Egypt.
[3] Chemistry Department, Faculty of Science, Kafrelsheikh University, 33516, Kafr ElSheikh, Egypt
[4] Department of Pharmaceutical Chemistry, College of Pharmacy, Jouf University, Sakaka, Kingdom of Saudi Arabia
[5] Department of Pharmaceutical Organic Chemistry, Faculty of Pharmacy, Beni-Suef University, Beni-Suef, Egypt
[6] Chemistry Department, College of Science, Jouf University, P.O. Box 2014, Sakaka, Saudi Arabia.
*Corresponding author at: Chemistry Department, College of Science, Taif University, P.O. box 11099, Taif 21944, Saudi Arabia. Tel.: +(966)543350861, E-mail address: ihelmy2003@yahoo.com, i.helmy@tu.edu.sa (I.H.E)

I- DFT Calculations

Figure S1: Optimized structure of **4** and **5** derivatives at B3LYP /6-311++G(d,p) level of theory in the gas phase.

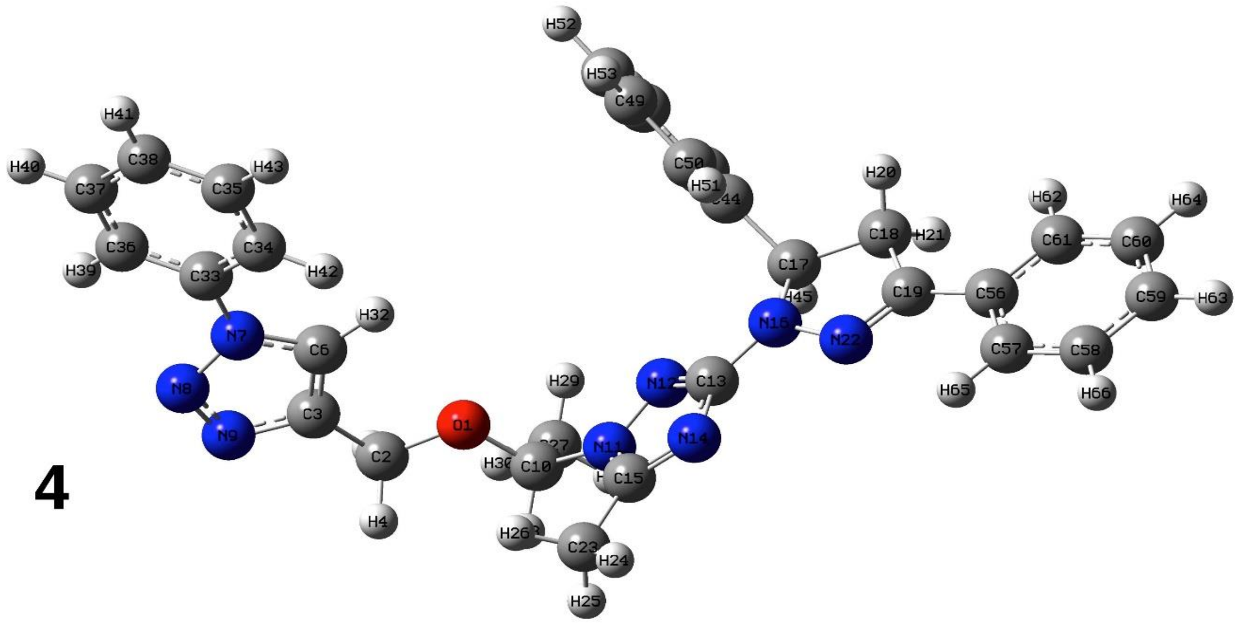

4

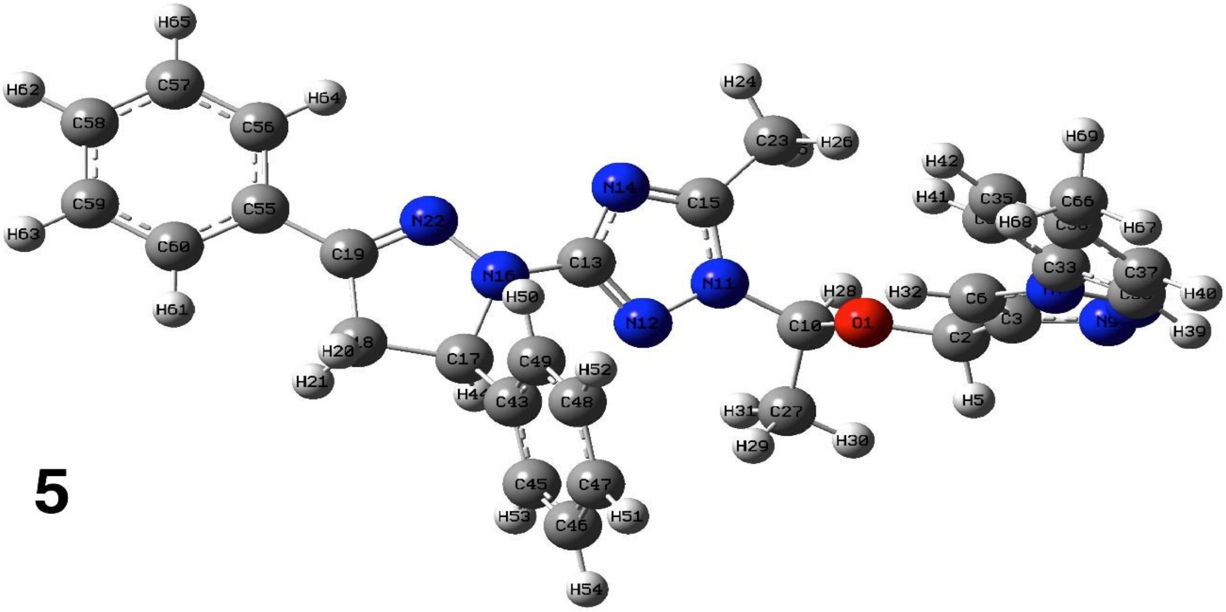

5

Figure S2: optimized structure of **6** and **7** derivatives at B3LYP/6-311++G(d,p) level of theory in the gas phase.

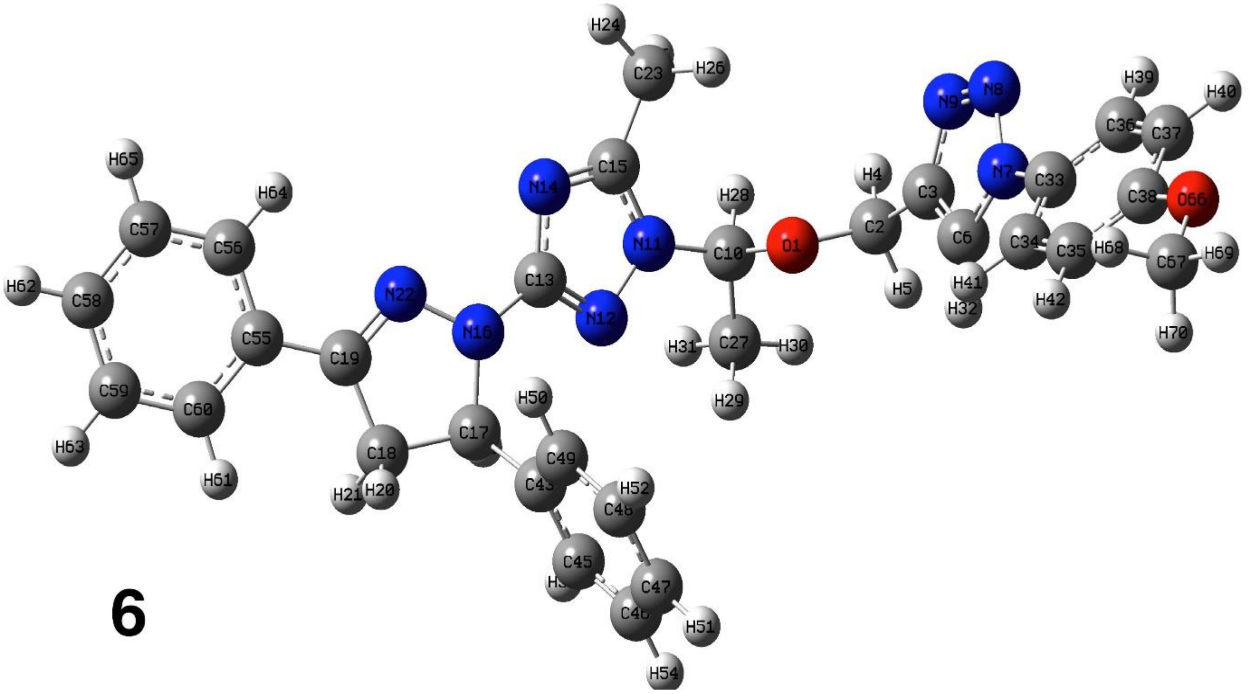

6

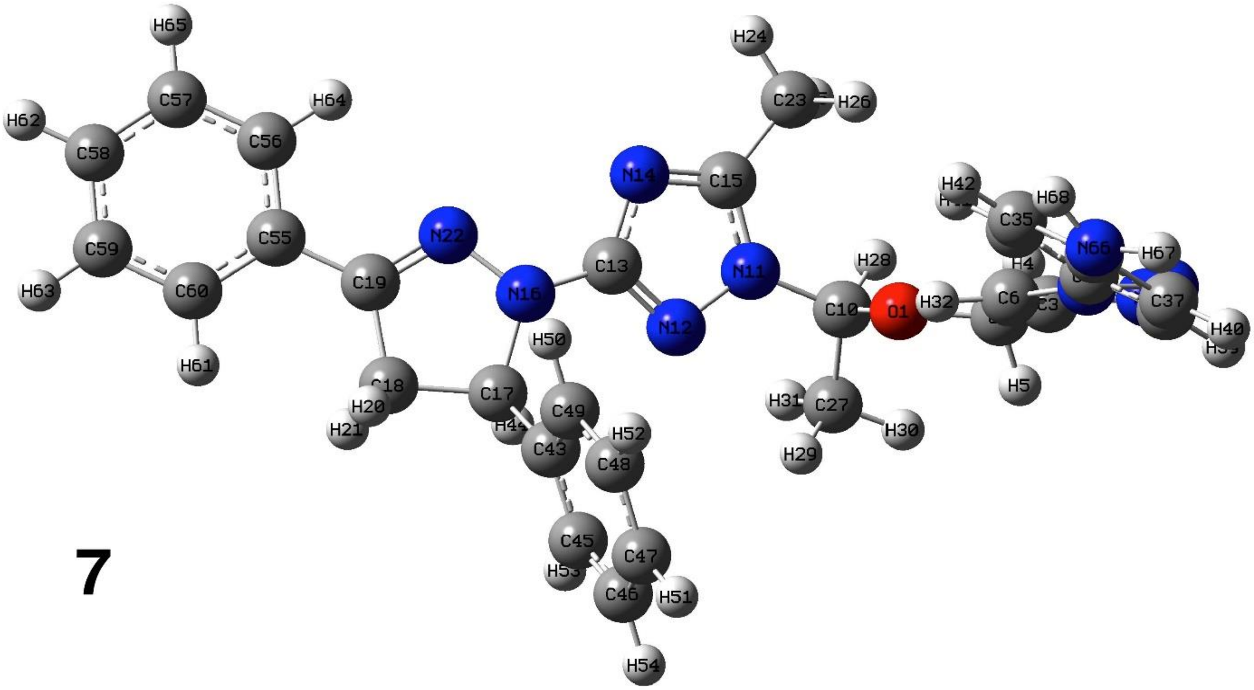

7

II- **Molecular docking data**

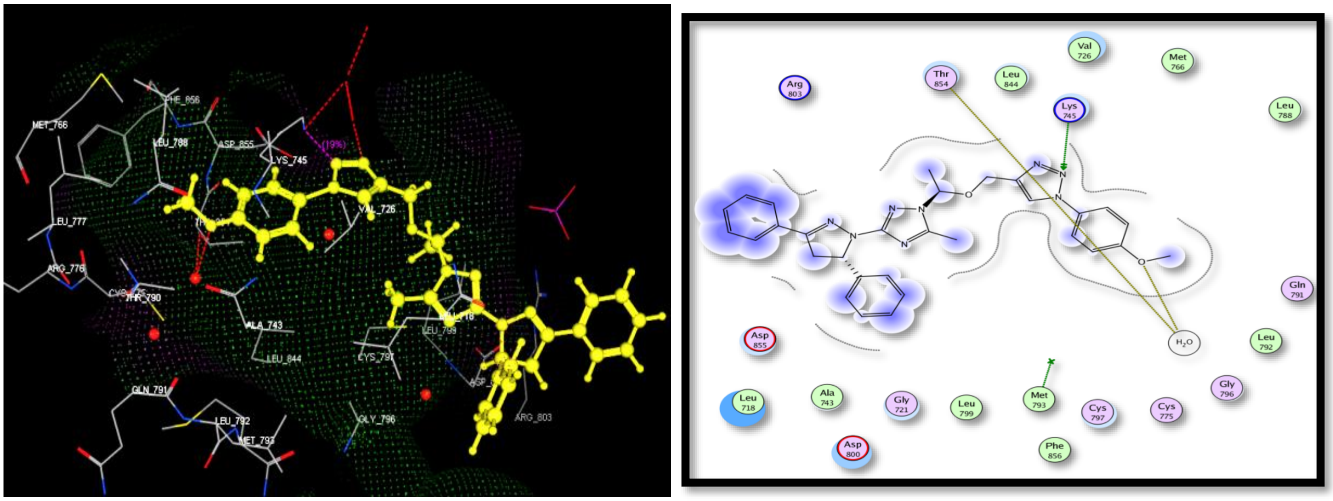

(A) **(B)**

Figure S3. Docking of compound **6** within ATP bind site of EGFR. A) 3D biding mode. B) 2D binding mode.

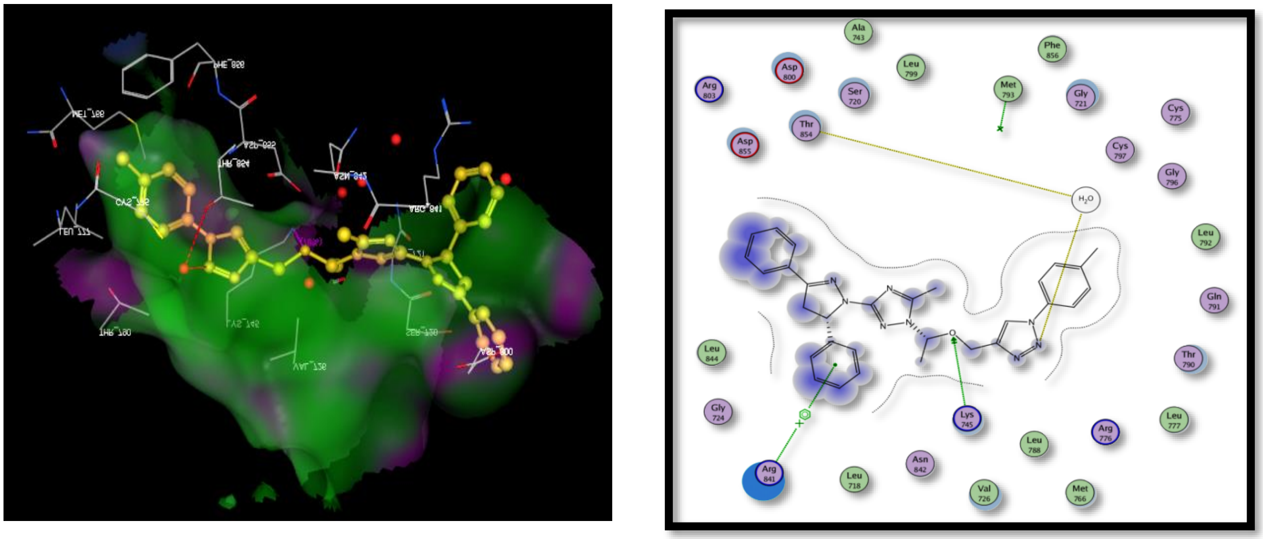

(A) (B)

Figure S4. Docking of compound **5** within ATP bind site of EGFR. A) 3D biding mode. B) 2D binding mode.

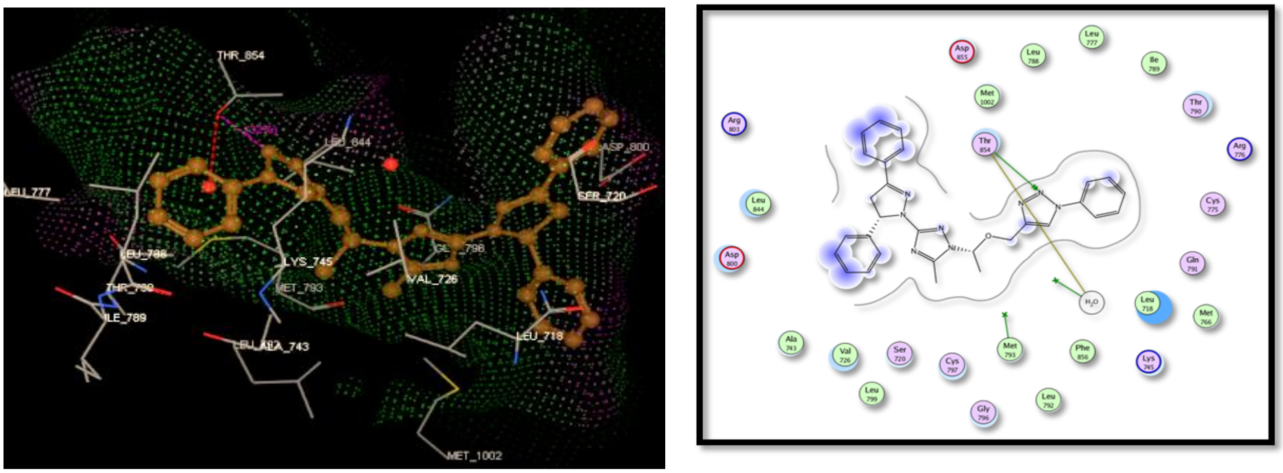

(A) (B)

Figure S5. Docking of compound **4** within ATP bind site of EGFR. A) 3D biding mode. B) 2D binding mode.

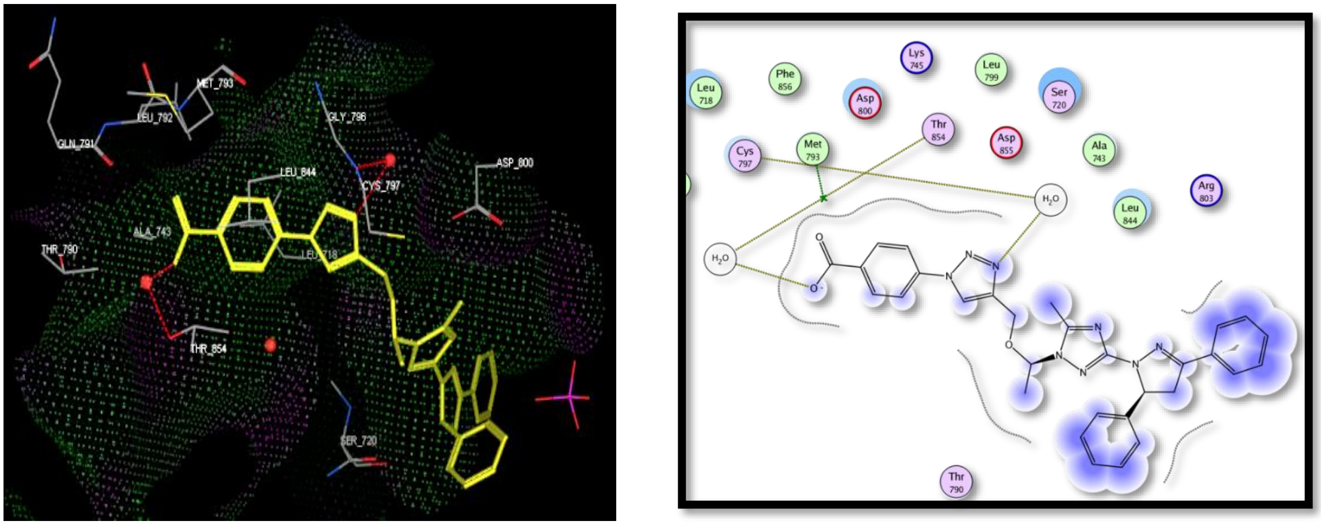

(A) (B)

Figure S6. Docking of compound **8** within ATP bind site of EGFR. A) 3D biding mode. B) 2D binding mode.

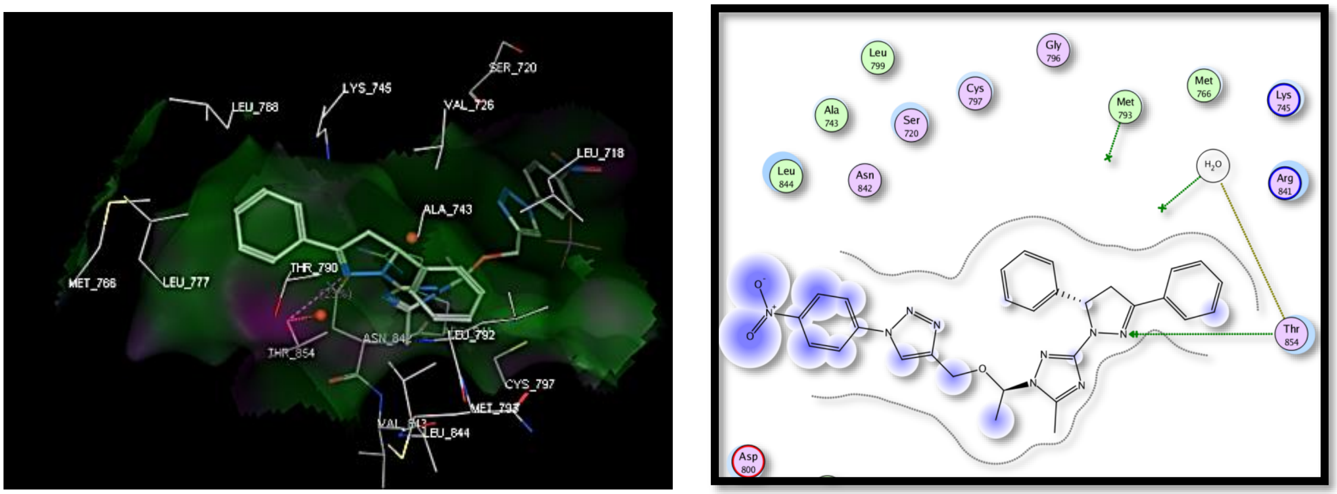

(A) (B)

Figure S7. Docking of compound **9** within ATP bind site of EGFR. A) 3D biding mode. B) 2D binding mode.

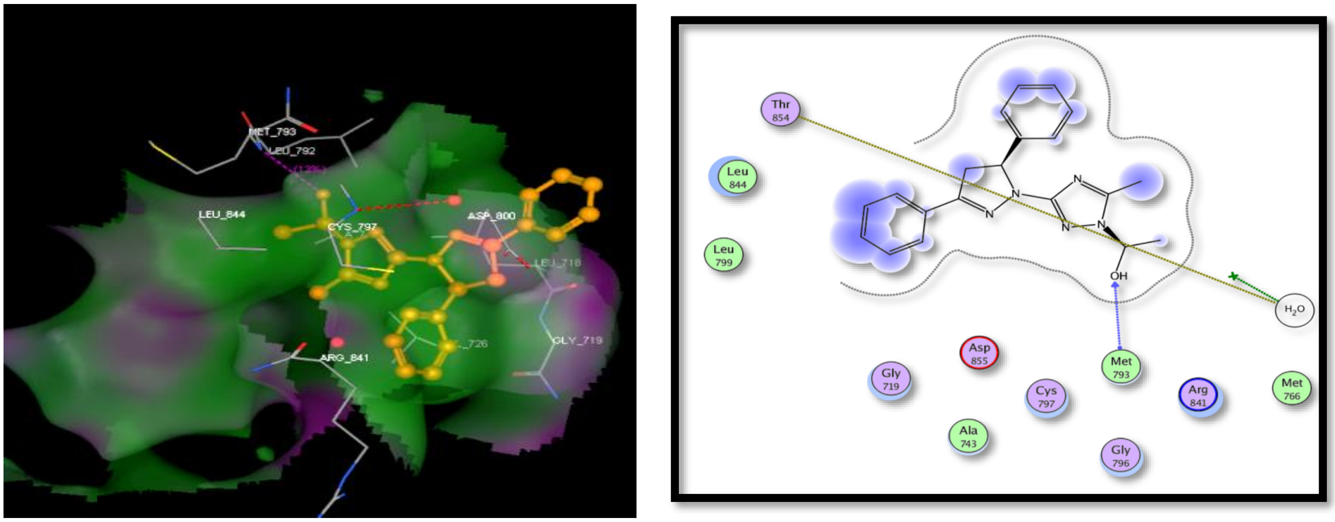

(A) (B)

Figure S8. Docking of compound **2** within ATP bind site of EGFR. A) 3D biding mode. B) 2D binding mode.

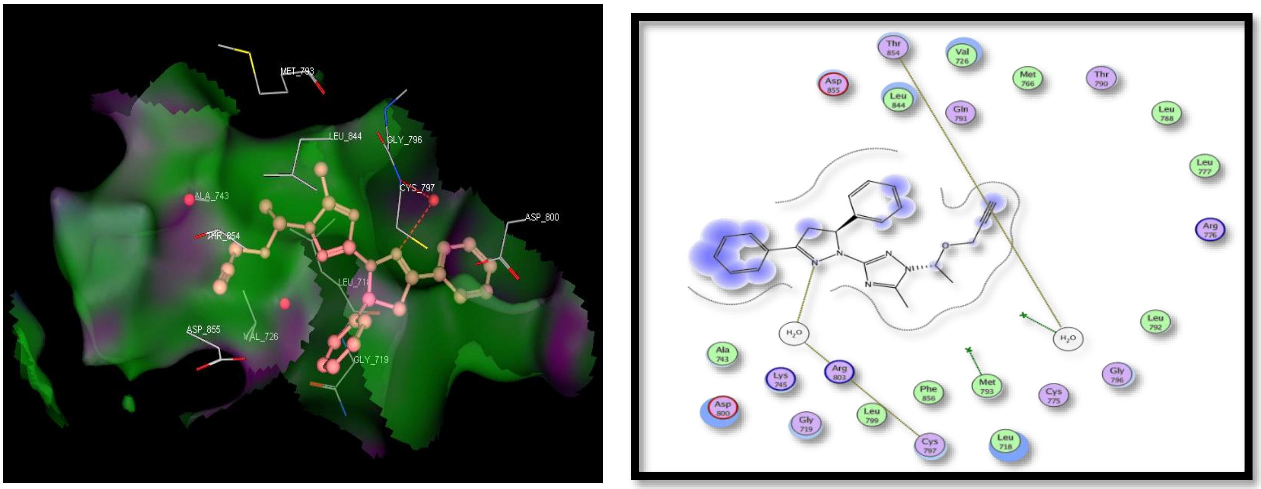

(A) (B)

Figure S9. Docking of compound **3** within ATP bind site of EGFR. A) 3D biding mode. B) 2D binding mode.

III. Spectra of synthesized compounds

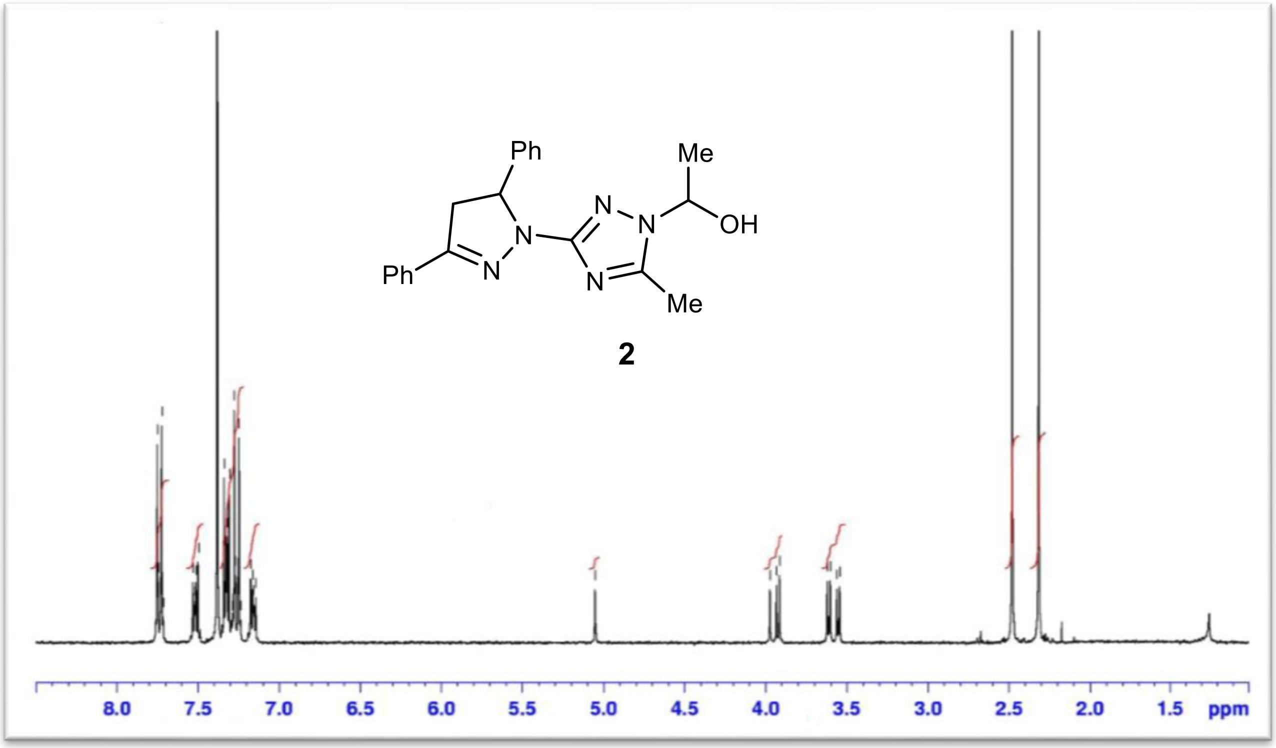

Figure S10. ^{1}H NMR of compound 2

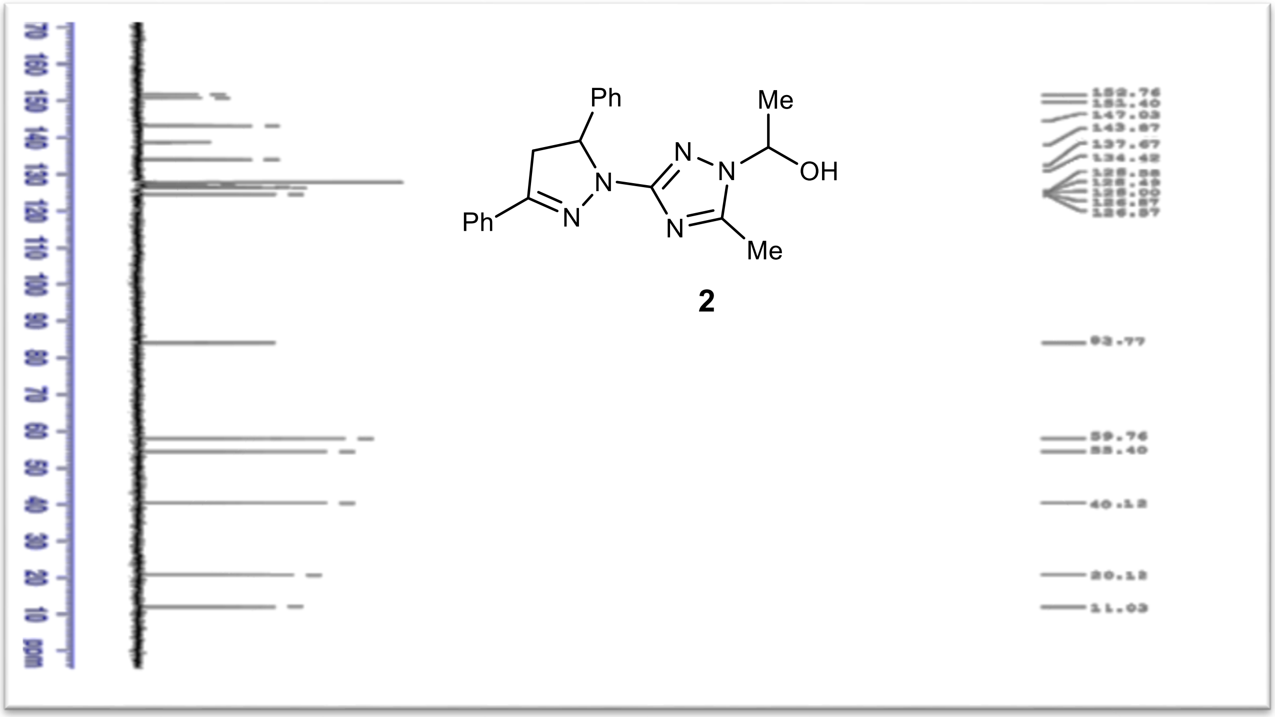

^{13}C NMR of compound 2

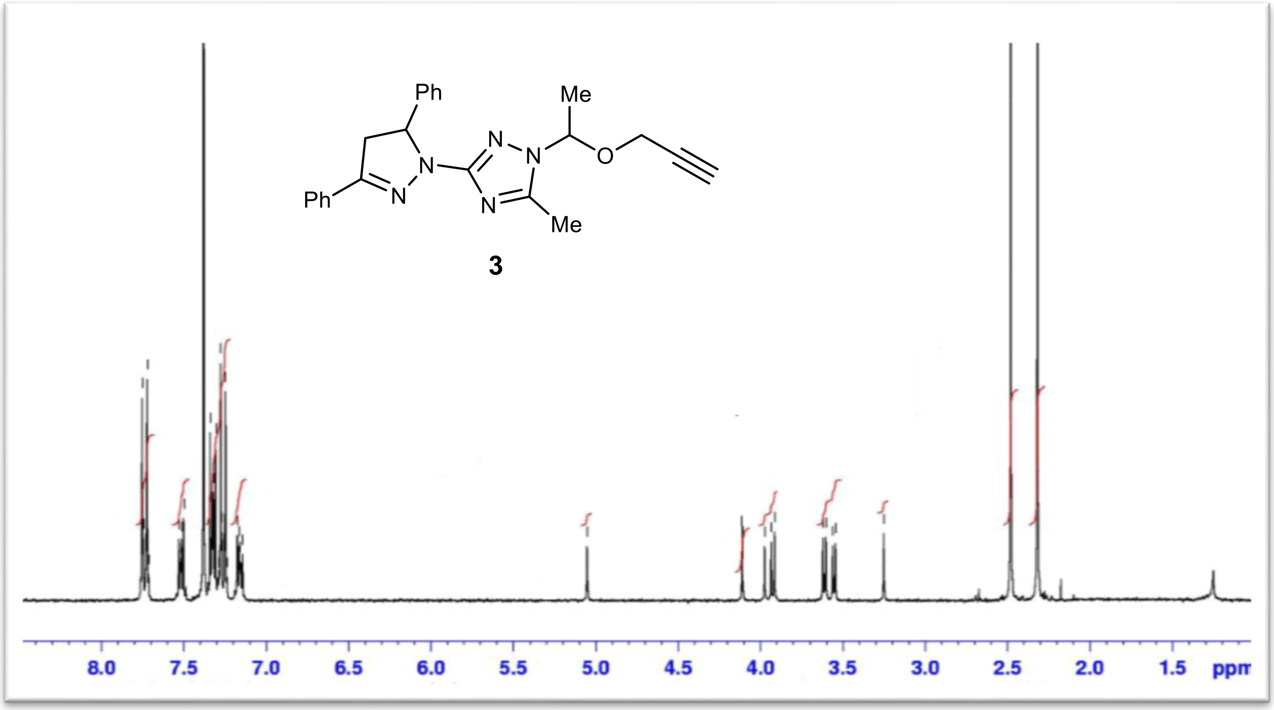

Figure S11. ^{1}H NMR of compound 3

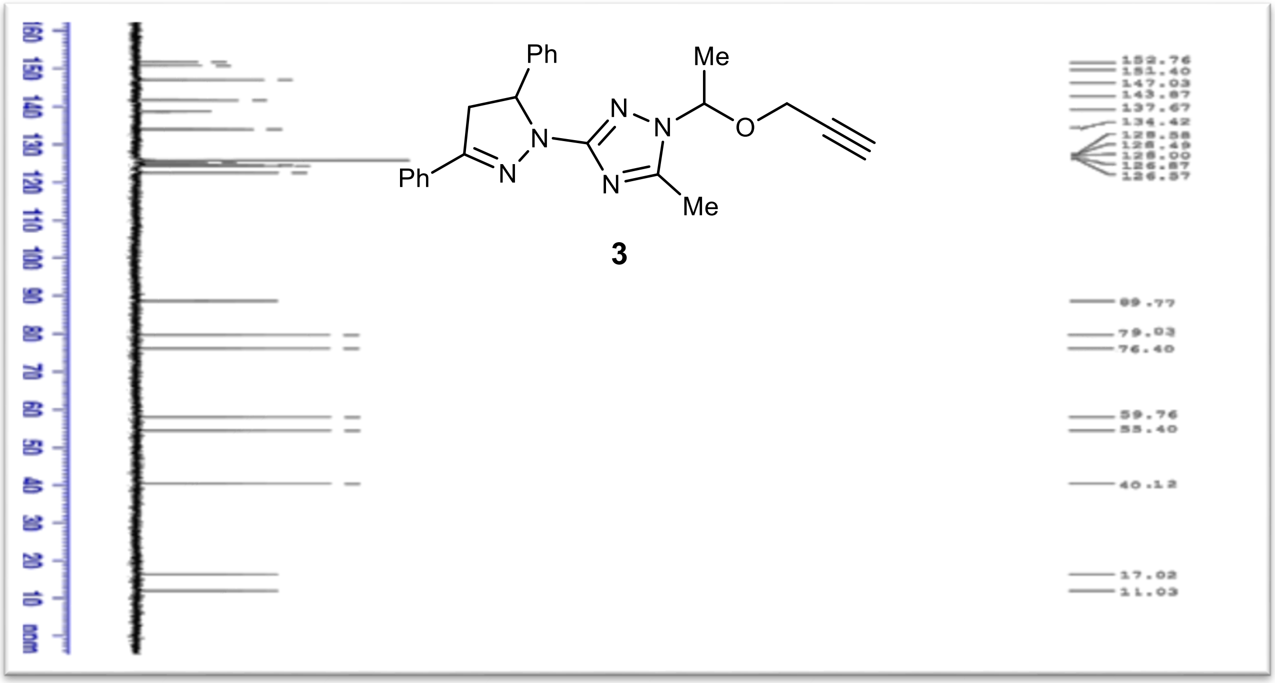

Figure S12. ^{13}C NMR of compound 3

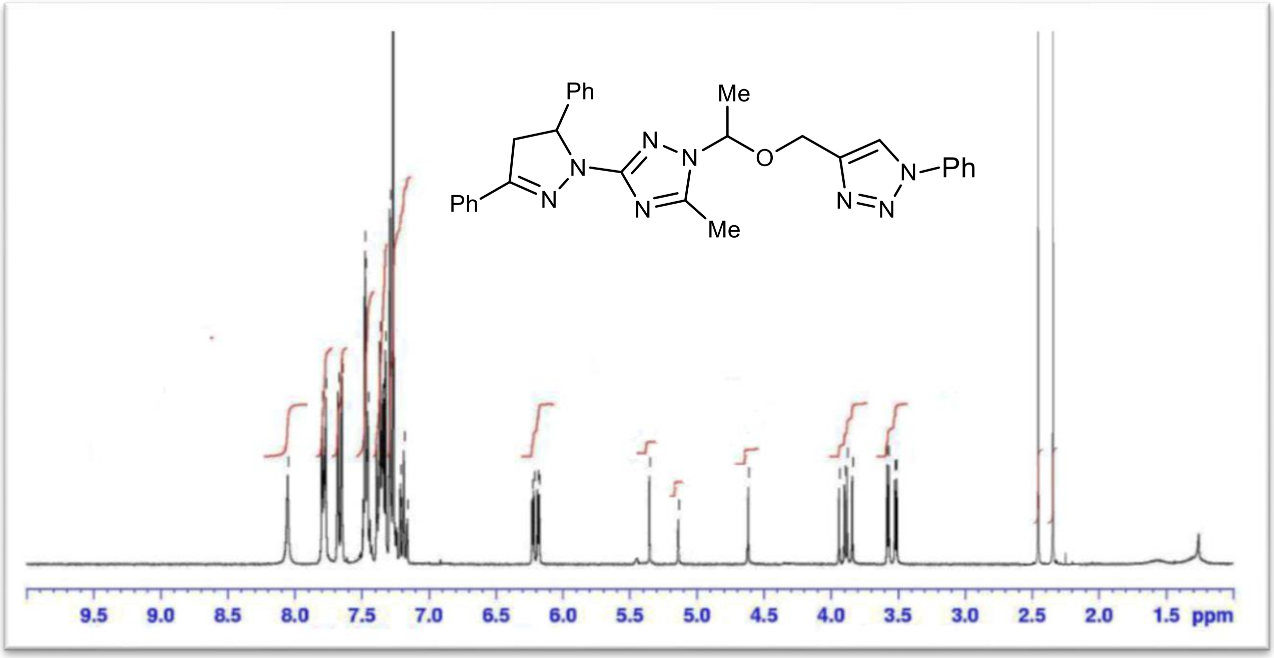

Figure S13. 1H NMR of compound 4

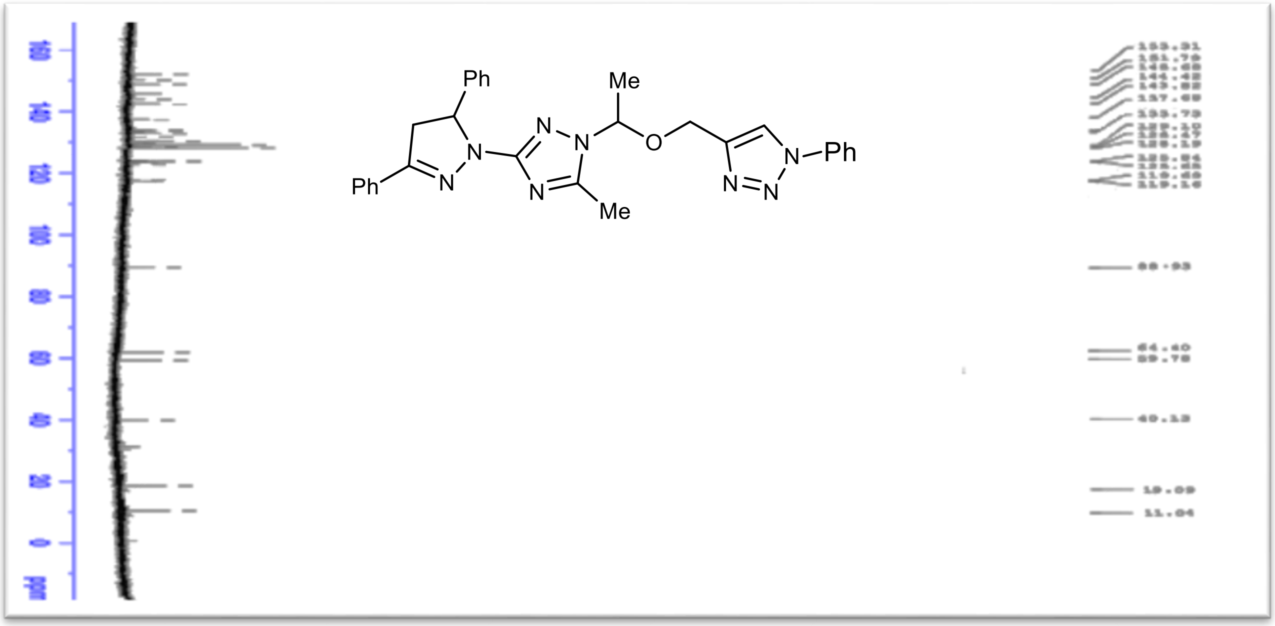

Figure S14. ^{13}C NMR of compound 4

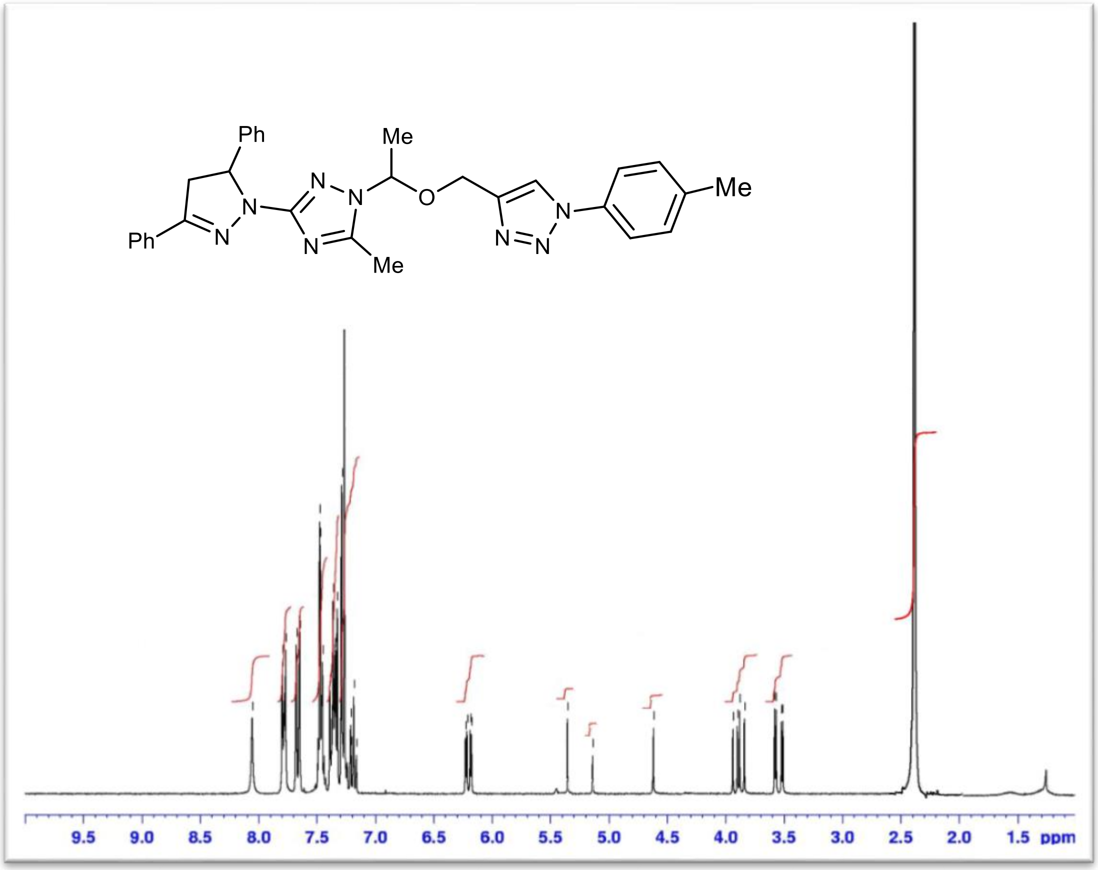

Figure S15. ^{1}H NMR of compound 5

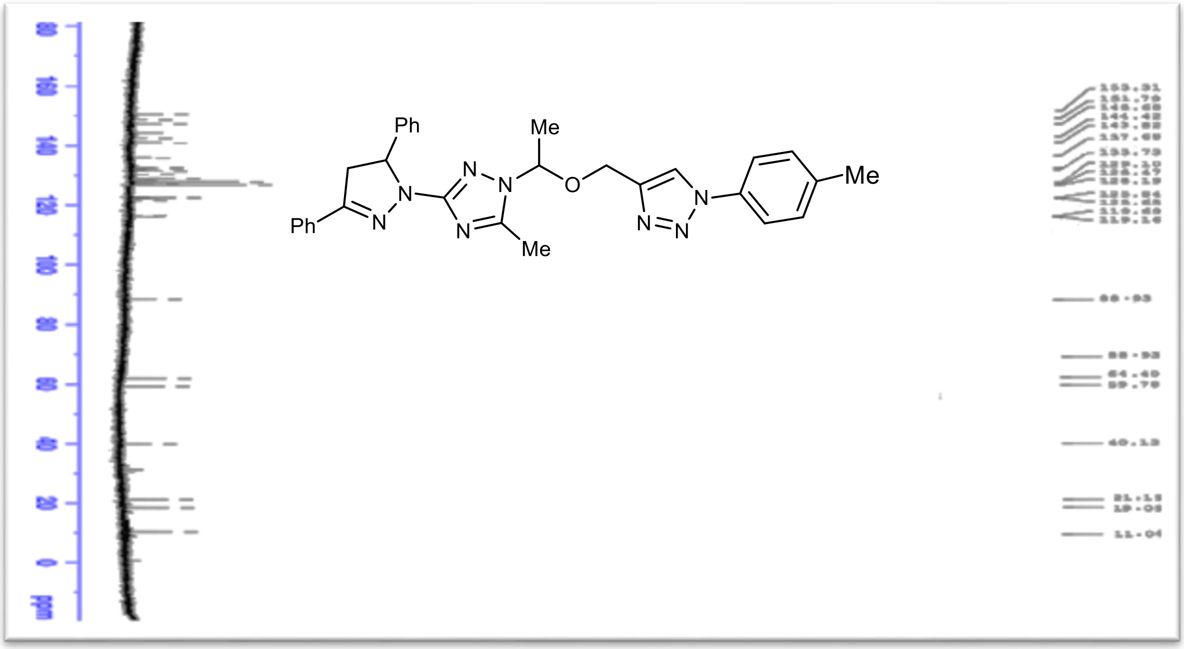

Figure S16. ^{13}C NMR of compound 5

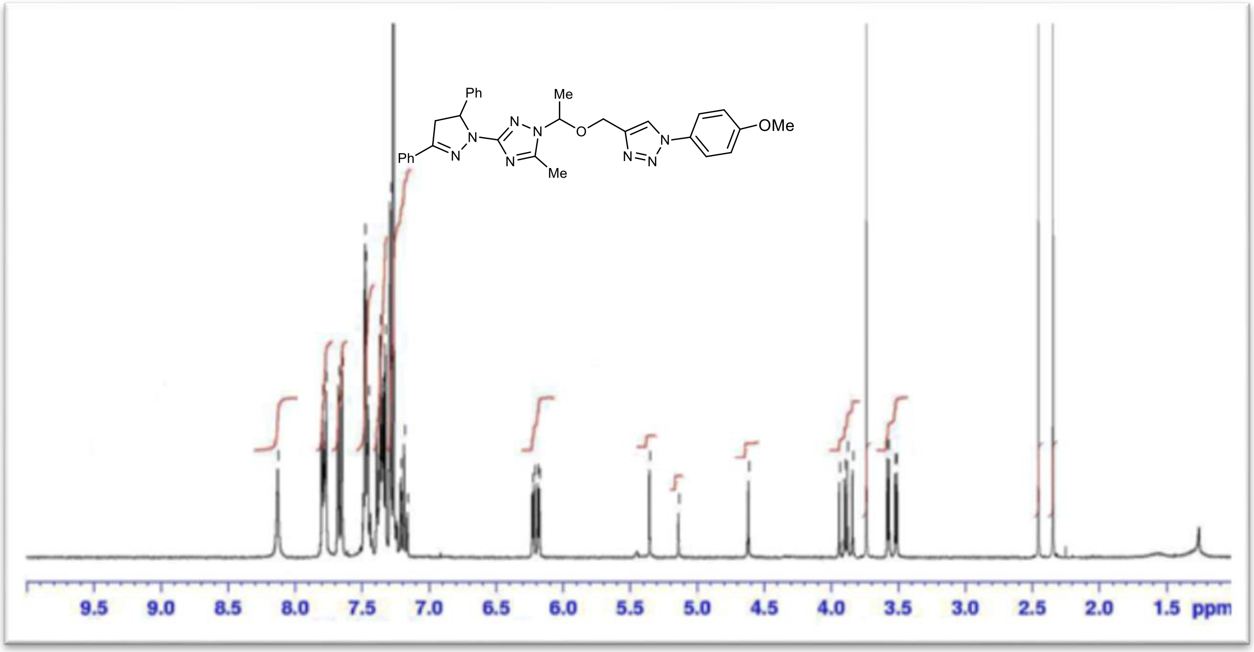

Figure S17. ^{1}H NMR of compound 6

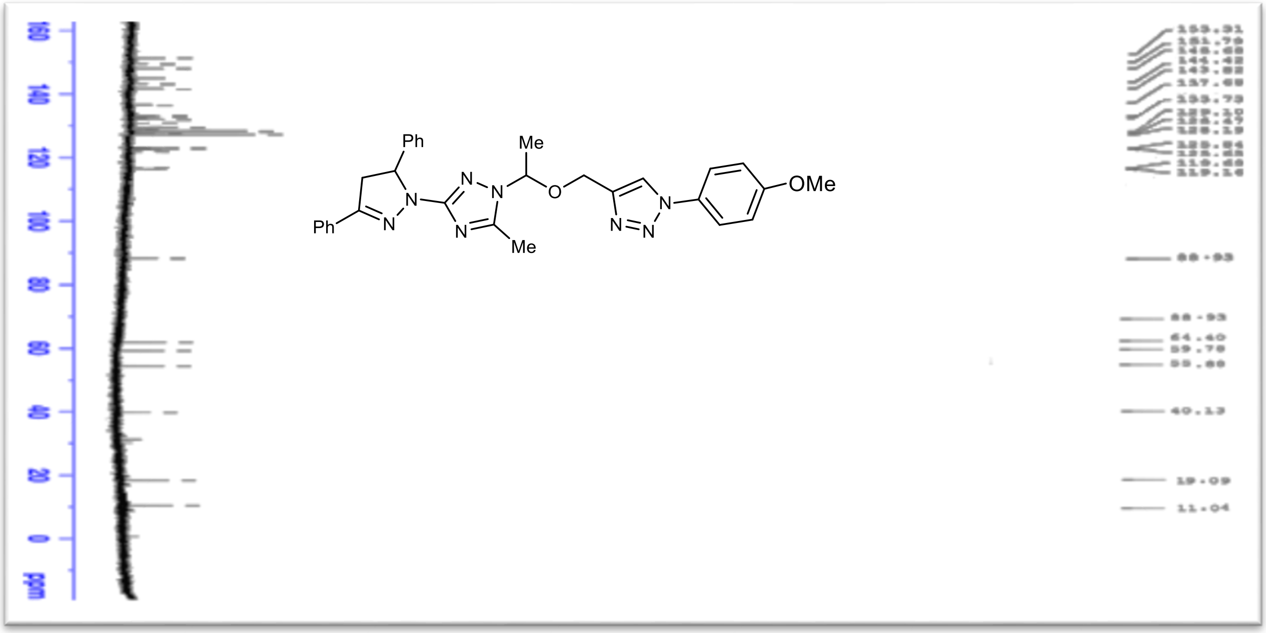

Figure S18. ^{13}C NMR of compound 6

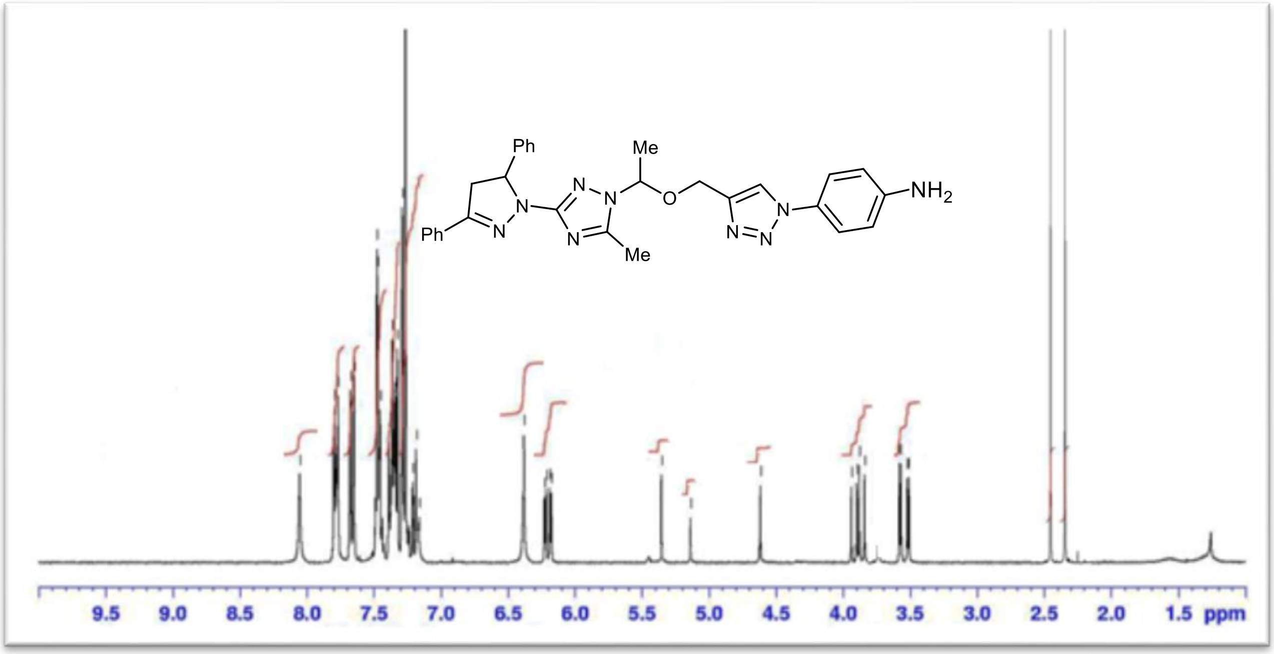

Figure S19. ^{1}H NMR of compound 7

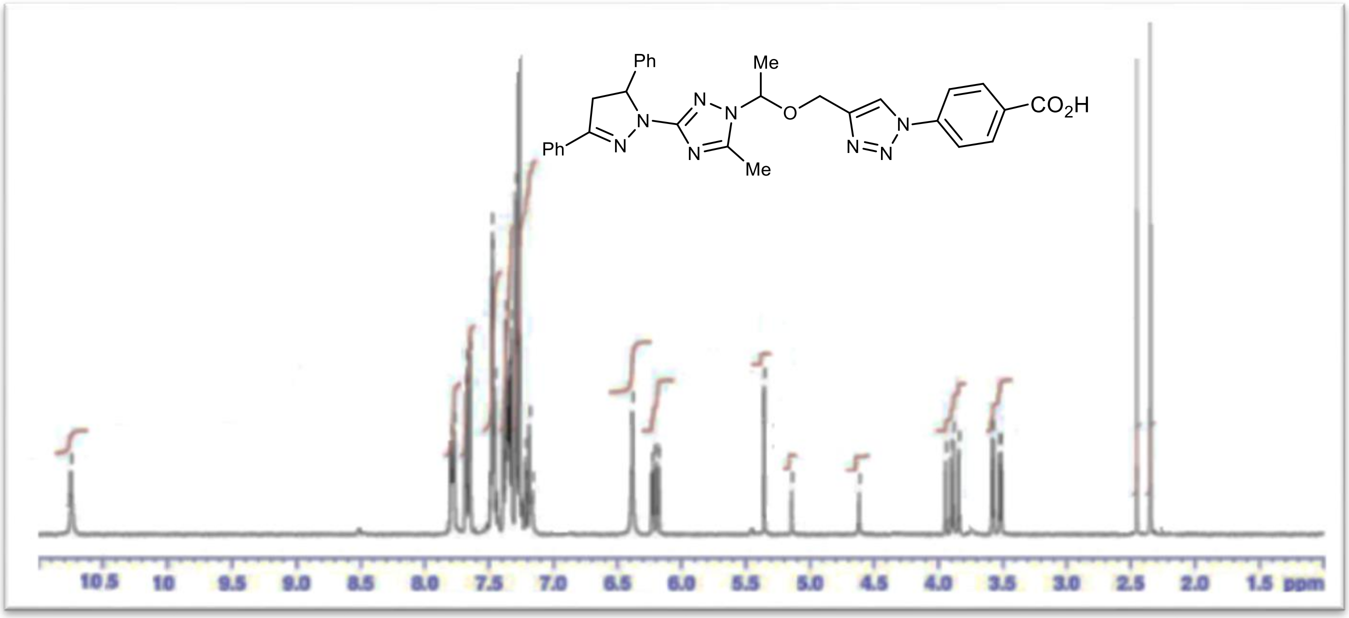

Figure S20. ^{1}H NMR of compound 8

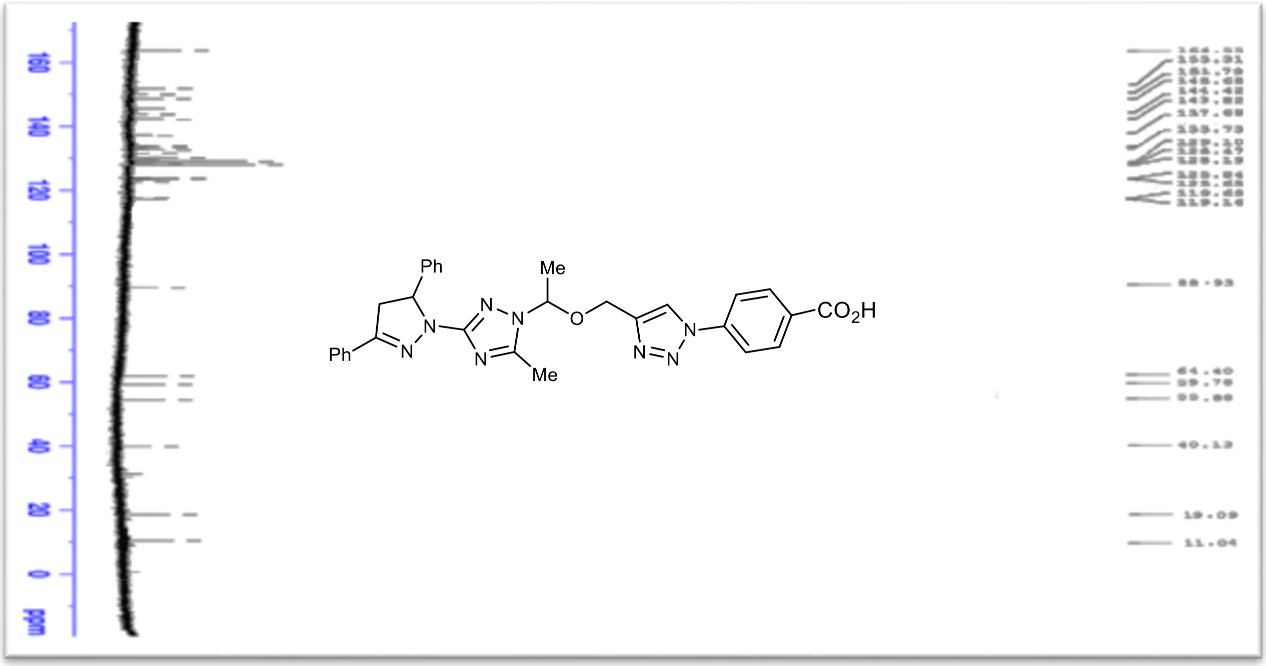

Figure S21. ^{13}C NMR of compound 8

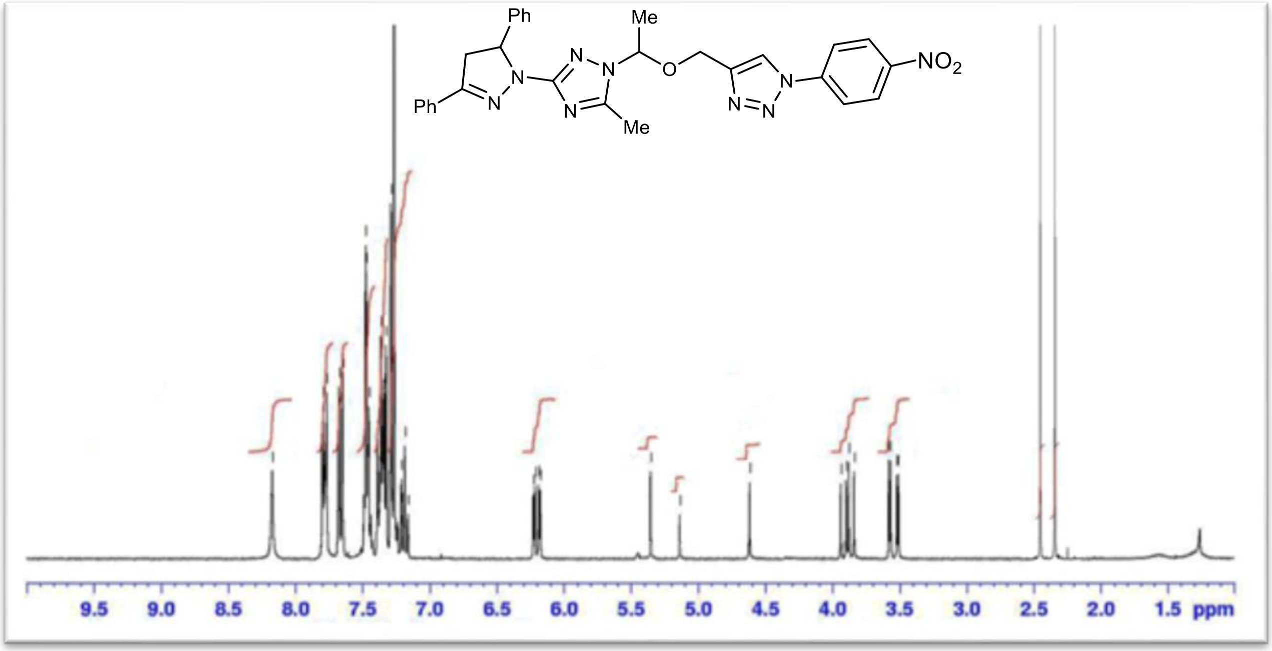

Figure S22. ^{1}H NMR of compound 9
